# Supplementary material for: Rho1 and Rgf1 establish a new actin-dependent signal to determine growth poles in yeast independently of microtubules and the Tea1–Tea4 complex
Source: PLoS Biol. 2024 Nov 7;22(11):e3002491. doi: 10.1371/journal.pbio.3002491 (PMC11602027; doi:10.1371/journal.pbio.3002491)
Supplement: S1 Table — (DOCX) [file pbio.3002491.s001.docx]

| **S1 Table. List of yeast strains used in this study** | | |
| --- | --- | --- |
| **Genotype** | **Source** | **Strain** |
| *leu1-32 his3d1 ura4d18 ade6M210 h^-^* | Our collection | MS38 |
| *rgf1::his3^+^ his3d1 ura4d18 leu1-32 ade6M210 h^-^* | Our collection | VT14 |
| *rgf1::kanMX6 leu1-32 ura4d18 ade6M210 h^-^* | Our collection | PG65 |
| *rgf1::his3^+^ his3d1 leu1-32 ade6M210 ura4d18 rgf1-GFP:leu1^+^ h^-^* | Our collection | PG40 |
| *crn1GFP:kanMX6 adeM210 leu1-32 ura4d18 h^+^* | Our collection | JCR962 |
| *mCherry-atb2:hphMX6, leu1-32, ura4d18, h-* | Phong T. Tran | TP2927 |
| *tea4-GFP:kanMX6 leu1-32 ura4d18 h^+^* | (1) | YSM210 |
| *tea4-GFP:kanMX6 rgf1::kanMX6 leu1-32 ade6M210 ura4d18* | This study | PG329 |
| *tea4-GFP:KanMX6, sad1:dsRed:natMX6, leu1-32, ura4d18, h^-^* | This study | TE136 |
| *aur:mCherry-atb2 tea4-GFP:kanMX6 leu1-32 ura4d18* | This study | PG353 |
| *aur:mCherry-atb2 tea4-GFP:kanMX6 rgf1::kan leu1-32 ura4d18* | This study | PG381 |
| *tea1-tomato:aur tea4-GFP:kanMX6 rgf1::his3^+^ his3d1 leu1-32* | This study | PG415 |
| *GFP-atb2:kanMX6* | (2) | MS1442 |
| *GFP-atb2:kanMX6 rgf1::kanMX6* | This study | PG497 |
| *mCherry-atb2:hphMX6, leu1-32 ura4d18 h^+^* | Sergio Rincón | JP24 |
| *rgf1::natMX6 mCherry-atb2:hphMX6 leu1-32 ura4d1 h^+^* | This study | PG545 |
| *tea1::ura4^+^ mCherry-atb2:hphMX6 leu1-32 ura4d1* | This study | PG547 |
| *tea4-GFP:kanMX6 mCherry-atb2:hphMX6 mod5::kanMX6 leu1-32* | This study | PG695 |
| *tea4-GFP:kanMX6 mCherry-atb2:hphMX6 rgf1::natMX6 mod5::kanMX6 ura4d1* | This study | PG580 |
| *tea1::ura4^+^ ura4d18 h^-^* | (3) | PN1687 |
| *mod5::kanMX6 leu1-32 ura4d18 ade6M210 h^+^* | This study | PG395 |
| *mod5::kanMX6 rgf1::kanMX6 leu1-32 ura4d18 ade6M210* | This study | PG404 |
| *cdc11-119 tea1::ura4^+^ leu1-32* | This study | PG212 |
| *cdc11-119 rgf1::kanMX6 leu1-32 h^-^* | This study | PG213 |
| *cdc11-119 mod5::kanMX6 leu1-32* | This study | PG721 |
| *cdc11-119 mod5::kanMX6 rgf1::natMX6 leu1-32* | This study | PG707 |
| *rgf1-HA:leu1^+^ rgf1::his3^+^ his3d1 ura4d18 leu1-32 ade6M210 h^-^* | Our collection | PG41 |
| *tea4-GFP:kanMX6 rgf1-HA:leu1^+^ rgf1::his3^+^ his3d1 leu1-32* | This study | PG402 |
| *tea1-GFP:kanMX6 ade6M210 leu1-32 ura4d18 h^-^* | (4) | KS1259 |
| *rgf1-ΔPH2-GFP:leu1^+^ rgf1::natMX6* | This study | PG650 |
| *rgf1-GFP:leu1^+^ rgf1::natMX6 efr3::kanMX6* | This study | PG653 |
| *tea4-GFP:kanMX6 rgf1::his3^+^ rgf1-ΔPH2:leu1^+^ his3d1 leu1-32 ura4d18 ade6M210 h^-^* | This study | PG705 |
| *tea4-GFP:kanMX6 rgf1-ΔPTTR:leu1^+^ rgf1::his3^+^ leu1-32 ura4d18 ade6M210 his3d1* | This study | PG378 |
| *rgf1::natMX6 rgf1-ΔPH2:leu1^+^ leu1-32 ura4d18 h^-^* | This study | PG703 |
| *rgf1::his3^+^ his3d1 leu1-32 ade6M210 ura4d18 rgf1ΔPTTR-GFP:leu1^+^ h^-^* | Our collection | PG199 |
| *pact1-LifeAct:GFP:leu1^+^, ura4d18, h^-^* | Our collection | JCR5240 |
| *tea1::ura4^+^ rgf1::his3^+^ ura4d18 his3d1* | This study | PG117 |
| *mod5::kanMX6 tea1::ura4 ura4d18* | This study | PG726 |
| *mEGFP-mod5 h^+^* | (5) | JM2888 |
| *pact1-LifeAct-mCherry:leu1^+^ rgf1::rgf1-GFP:kanMX6 ura4d18 leu1-32 h^+^* | This study | PG740 |
| *gef1::kanMX6 ura4d18 leu1-32 h^+^* | Pilar Pérez | PPG2517 |
| *gef1::kanMX6 tea1::ura4^+^* | This study | PG753 |
| *pact1-LifeAct-mCherry:leu1^+^ rgf1::rgf1-GFP:kanMX6 tea1::ura4^+^ ura4d18 leu1-32* | This study | PG742 |
| *tea1-GFP:kanMX6 rgf1::his3+ his3d1 leu1-32 ura4d18 h^+^* | This study | PG385 |
| *tea1-GFP:kanMX6 Mod5::kanMX6 h^-^* | This study | PG552 |
| *tea1-GFP:kanMX6 Mod5::kanMX6 rgf1::natMX6 h^-^* | This study | PG760 |
| *tea4-GFP:kanMX6 cdc10 leu1-32* | This study | PG785 |
| *tea4::kanMX6 leu1-32 ura4d18 ade6M210 h^+^* | (1) | YSM144 |
| *cdc11-119 tea4::kanMX6 leu1-32* | This study | PG762 |
| *rgf1-ΔPH2-ΔPTTR:leu1^+^ tea4-GFP:kanMX6 rgf1::his3^+^ his3d1 leu1-32 ura4d18 ade6M210 h^-^* | This study | PG791 |
| *pact1-rgf1-ΔPH2-GFP:leu1^+^ rgf1::natMX6 leu1-32 ura4d18 h^-^* | This study | PG793 |
| *tea4-GFP:kanMX6 pact1-rgf1-ΔPH2:leu rgf1::natMX6 leu1-32* | This study | PG804 |
| *CRIB-GFP:ura4^+^ rgf1::kanMX6* | This study | PG331 |
| *CRIB-GFP: ura4^+^ tea4-GBP-cherry:kanMX6 rgf1::kanMX6* | This study | RC627 |
| *tea4::kanMX6 rgf1-GFP:leu rgf1::his3^+^, h^+^* | This study | PG342 |
| *for3::kanMX6 mx1 leu1-32 ura4d-18 ade6 h^-^* | (6) | BFY9 |
| *sty1-GFP:kanMX6 leu1-32 h^-^* | (7) | EH005 |
| *sty1-GFP:kanMX6 rgf1::natMX6 leu1-32 h^-^* | This study | PG774 |
| *pact1-LifeAct-mCherry:leu rgf1::rgf1-GFP:kanMX6 sty1::natMX6 ura4d18 leu1-32 h^+^* | This study | PG754 |
| *tea1::ura4^+^ rgf1::his3^+^ sty1::kanMX6 ura4d18 his3d1* | This study | PG780 |
| *S. cerevisiae: MATa, trp1-901, leu2-3, 112, ura3-52, his3-200, gal4Δ, gal80Δ, LYS2::GAL1UAS-GAL1TATA-HIS3, GAL2UAS-GAL2TATA-ADE2, URA3::MEL1UAS-MEL1 TATA-lacZ* | Clontech (Takara, Mountain View, CA) | AH109 |

1. Martin SG, McDonald WH, Yates 3rd JR, Chang F. Tea4p links microtubule plus ends with the formin for3p in the establishment of cell polarity. Dev Cell [Internet]. 2005;8(4):479–91. Available from: https://www.ncbi.nlm.nih.gov/pubmed/15809031

2. Sato M, Toda T. Alp7/TACC is a crucial target in Ran-GTPase-dependent spindle formation in fission yeast. Nature. 2007 May 2;447(7142).

3. Mata J, Nurse P. tea1 and the microtubular cytoskeleton are important for generating global spatial order within the fission yeast cell. Cell [Internet]. 1997;89(6):939–49. Available from: https://www.ncbi.nlm.nih.gov/pubmed/9200612

4. Snaith H, Sawin KE. Fission yeast mod5p regulates polarized growth through anchoring of tea1 at the cell tips. Nature. 2003;423:647–51.

5. Kettenbach AN, Deng L, Wu Y, Baldissard S, Adamo ME, Gerber SA, et al. Quantitative Phosphoproteomics Reveals Pathways for Coordination of Cell Growth and Division by the Conserved Fission Yeast Kinase Pom1*. Molecular & Cellular Proteomics. 2015 May;14(5).

6. Feierbach B, Chang F. Roles of the fission yeast formin For3 in cell polarity, actin cable formation and symmetric cell division. Curr Biol. 2001;11:1656–65.

7. Zuin A, Vivancos AP, Sansó M, Takatsume Y, Ayté J, Inoue Y, et al. The Glycolytic Metabolite Methylglyoxal Activates Pap1 and Sty1 Stress Responses in Schizosaccharomyces pombe. Journal of Biological Chemistry. 2005 Nov;280(44).
