# Supplementary material for: Rho1 and Rgf1 establish a new actin-dependent signal to determine growth poles in yeast independently of microtubules and the Tea1–Tea4 complex
Source: PLoS Biol. 2024 Nov 7;22(11):e3002491. doi: 10.1371/journal.pbio.3002491 (PMC11602027; doi:10.1371/journal.pbio.3002491)
Supplement: S2 Table — (DOCX) [file pbio.3002491.s002.docx]

| **S2 Table. List of plasmids used in this study.** | | | |
| --- | --- | --- | --- |
| **Name** | **Description** | **Selection markers** | |
| **Two-hybrid vectors** | | | |
| pGR135 | pGAD-*tea1* | amp | LEU2 |
| pGR106 | pGAD-*tea4* | amp | LEU2 |
| pRZ97 | pGBK-*rgf1* | kan | TRP1 |
| **Bacterial expression vectors** | | | |
| pGR152 | pGEX2T-GST-*rgf1* | amp |  |
| pGR129 | pGEX2T-GST-*tea4* | amp |  |
| pGR128 | pGEX2T-GST-*rgf1ΔCNH1* | amp |  |
| pGR138 | pGEX2T-GST-*rgf1ΔPH-CNH1* | amp |  |
| pGEX-C21RBD | pGEX2T-GST-C21RBD | amp |  |
| ***S. pombe* expression vectors** | | | |
| pREP4x-Rho1 | pREP4x-HA-*rho1* | amp | *ura4* |
| pGR100 | pAL-*rgf1-tomato* | amp | LEU2 |
| pGR145 | pIJ148-*rgf1ΔPH-GFP* | amp | LEU2 |
| pGR161 | pIJ148-*pact-rgf1ΔPH-GFP* | amp | LEU2 |
| pGR1159 | pIJ148-*rgf1ΔPH-ΔPTTR* | amp | LEU2 |
