# Supplementary figures and images for: Rho1 and Rgf1 establish a new actin-dependent signal to determine growth poles in yeast independently of microtubules and the Tea1–Tea4 complex
Source: PLoS Biol. 2024 Nov 7;22(11):e3002491. doi: 10.1371/journal.pbio.3002491 (PMC11602027; doi:10.1371/journal.pbio.3002491)

Figure S1

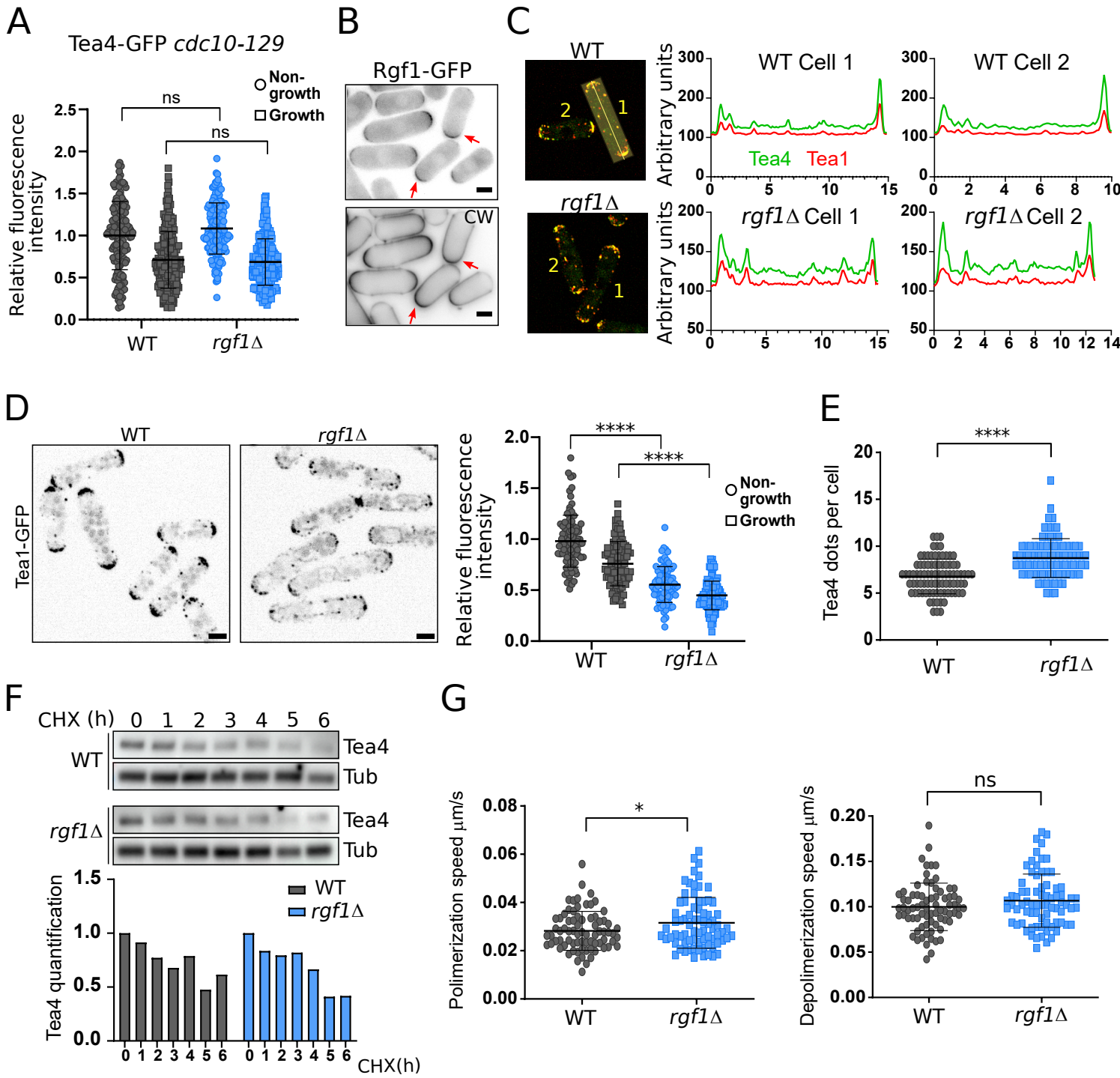

Supplement: S1 Fig — (A) The graphic represents the mean ± SD of the relative fluorescence intensity of Tea4-GFP measured at the growing and non-growing ends of WT cells (n > 150 for each end) and cdc10-129 cells (n > 150 for each end) grown for 4 h at 37°C. Calcofluor staining was used to differentiate the growing poles from the non-growing poles. (B) Wild-type cells expressing rgf1-GFP were stained with Calcofluor white (20 μg/ml) to spot areas of growth. The arrows indicate the localization of Rgf1 at the growing tip in monopolar cells. (C) Maximum projection images of the cells from Fig 1C show the merged localization of Tea4-GFP (green) and Tea1-tomato (red) in wild-type (WT) and rgf1Δ cells (left panels). The fluorescence intensity profiles for green and red channels along a line across the poles, covering the cell width, are shown for cells 1 and 2 of WT and rgf1Δ cells, respectively, in the right panels. Scale bar, 2 μm. (D) GFP fluorescence in cells expressing tea1-GFP rgf1+ and tea1-GFP rgf1Δ grown to log-phase at 28°C in YES medium. The maximum-intensity projection of 6 Z-slides (0.5 μm step-size) of Tea1-GFP fluorescence is shown. The graphic represents the mean ± SD of the relative fluorescence intensity of Tea1-GFP measured at the growing and non-growing ends of WT (n > 80 for each end) and rgf1Δ (n > 80 for each end) cells. (E) Quantitation of the number of Tea4 dots associated to the MTs in WT and rgf1Δ strains per cell. The mean ± SD of >80 cells is shown. (F) The WT and rgf1Δ cells expressing tea4-GFP were treated with the translation inhibitor cycloheximide (CHX, 100 μg/ml) for the indicated times. Proteins were visualized by western blot with antibodies against GFP (Tea4) or tubuline (Tub), as a loading control (upper). The graphic represents the quantification of Tea4 levels at different times (hours) after the treatment relative to time 0, which was assigned a value of 1 (bottom). (G) The graphics show the MT polymerization (left) and depolymerization (right) rates [file pbio.3002491.s003.pdf]

Figure S2

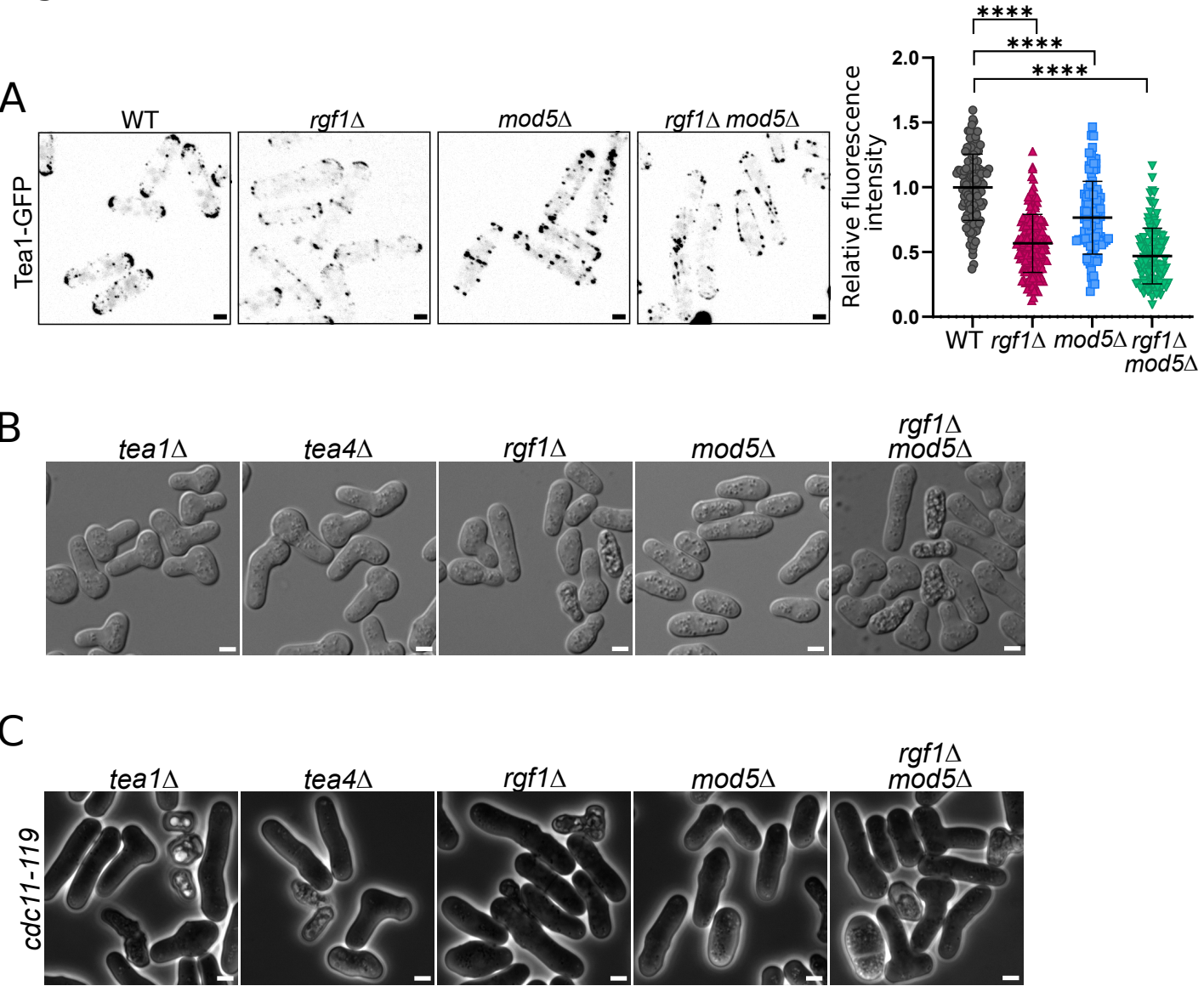

Supplement: S2 Fig — (A) Representative images of the indicated strains producing Tea1-GFP. The maximum-intensity projection of 6 Z-slides (0.5 μm step-size) is shown. Scale bar, 2 μm. The graph represents the mean ± SD of the relative fluorescence intensity of Tea1-GFP measured at the cell tips in the WT, rgf1Δ, mod5Δ, and rgf1Δ mod5Δ cells (n > 100). WT levels were used for normalization. (B) Cell morphology of the indicated strains after refeeding treatment (-MBC). (C) Cell morphology of the indicated strains after 4 h at 36°C in YES liquid medium. Scale bar, 2 μm. ****P < 0.0001. The data underlying the graphs shown in the figure can be found in S1 Data. (PDF) [file pbio.3002491.s004.pdf]

Figure S3

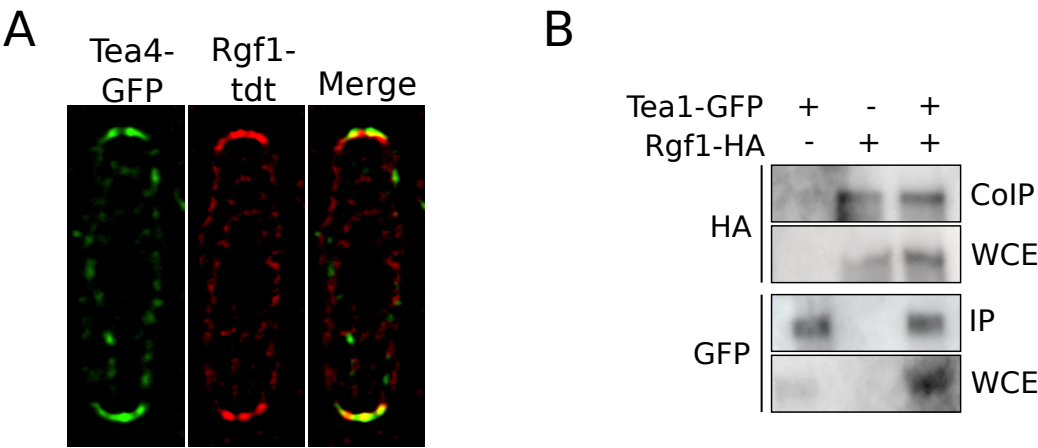

Supplement: S3 Fig — (A) Colocalization of Rgf1 and Tea4. Super-resolution radial fluctuations (SRRF) images of WT cells producing Tea4-GFP endogenously (green) and Rgf1-tdTomato from a plasmid under the control of its own promoter (red). (B) Coprecipitation of Rgf1 and Tea1. Cell extracts from cells producing Tea1-GFP, Rgf1-HA, and Tea1-GFP and Rgf1-HA were precipitated with GFP-trap beads and blotted with anti-HA or anti-GFP antibodies (co-immunoprecipitation and immunoprecipitation). Western blot was performed on total extracts to visualize total Tea1-GFP and Rgf1-HA levels (whole cell extracts). The data underlying the graphs shown in the figure can be found in S1 Data. (PDF) [file pbio.3002491.s005.pdf]

Figure S4

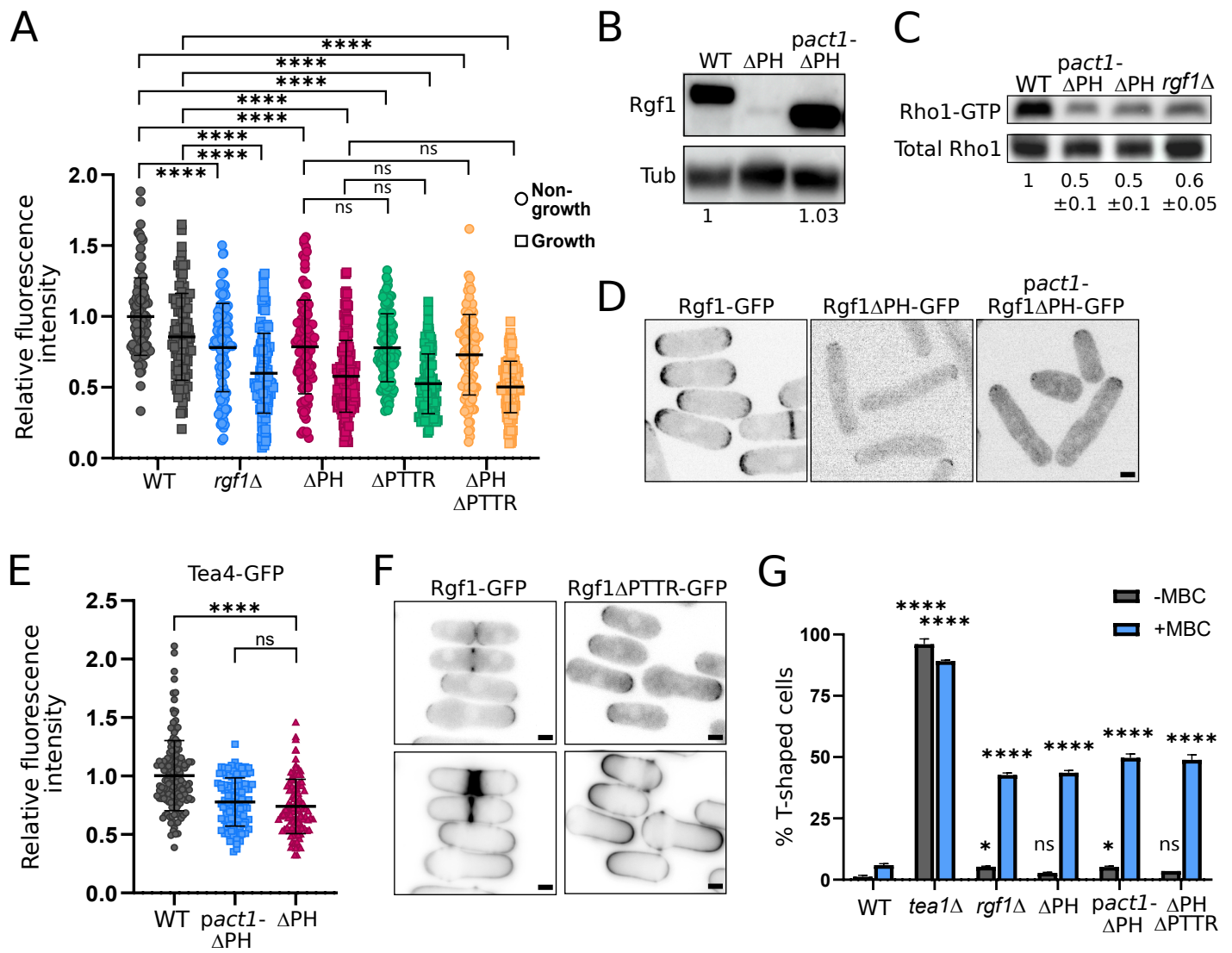

Supplement: S4 Fig — (A) The graphic represents the mean ± SD of the relative fluorescence intensity of Tea4-GFP measured at the growing and non-growing ends of the strains indicated (n > 100 for each end). Calcofluor staining was used to differentiate the growing poles from the non-growing poles. (B) Protein extracts from cells producing rgf1+-GFP, rgf1ΔPH-GFP, and pact-rgf1ΔPH-GFP were analyzed by western blot with an anti-GFP antibody to visualize Rgf1 levels. An anti-tubulin antibody was used as a loading control. (C) Extracts from cells producing Rho1-HA (pREP4X-Rho1-HA) in the WT, rgf1Δ, rgf1ΔPH, and pact-rgf1ΔPH-GFP cells were pulled down with GST-C21RBD and blotted against anti-HA antibody (Rho1-GTP). Total Rho1-HA was visualized by western blot (WCE). The relative units indicate the fold-differences in Rho1 levels in the mutants compared with the WT strain, with an assigned value of 1 (bottom) from 2 independent experiments. (D) Maximum-intensity projection of 6 Z-slides (0.5 μm step-size) of representative fluorescence images of WT, rgf1ΔPH-GFP, and pact-rgf1ΔPH-GFP cells. (E) The graphic represents the mean ± SD of the relative fluorescence intensity of Tea4-GFP (n > 120) measured at the cellular tips of the WT, rgf1ΔPH, and pact-rgf1ΔPH. Statistical significance was calculated using a two-tailed unpaired Student’s t test. (F) Representative images of Rgf1-GFP and Rgf1ΔPTTR-GFP localization. Cells were stained with calcofluor white (20 μg/ml) to spot areas of growth. Scale bar, 2 μm. (G) The percentage of cells WT, tea1Δ, rgf1Δ, rgf1ΔPH, pact-rgf1ΔPH, and rgf1ΔPH-ΔPTTR cells forming branches 3 h after release to growth after 3 days in stationary phase, in the absence and in the presence of MBC (50 μg/ml). The mean ± SD of >200 cells from 3 independent experiments is shown. Statistical significance of each strain compared to the WT was calculated using a two-way ANOVA test. ****P < 0.0001, *P < 0.05, ns = nonsignificant. The data underlying the graphs shown in the figure can b [file pbio.3002491.s006.pdf]

Figure S5

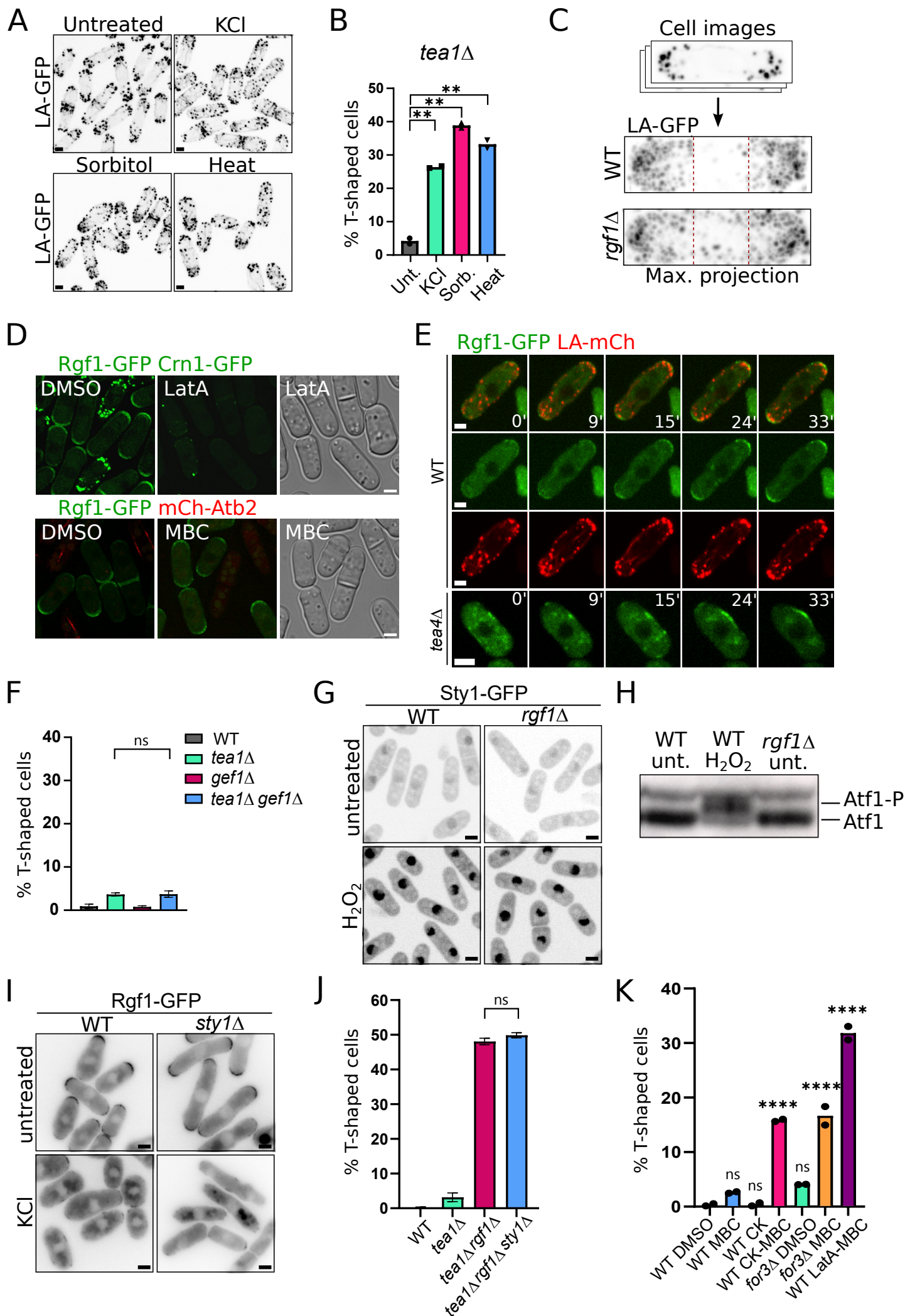

Supplement: S5 Fig — (A) Representative images of LifeAct-GFP (actin) localization in WT cells untreated or treated with KCl 0.6 M, sorbitol 1.2 M, or 37°C (heat) for 1 h. The maximum-intensity projection of 6 Z-slides (0.5 μm step-size) of fluorescence is shown. (B) Quantitation of the T-shaped cells in the tea1Δ mutant treated with DMSO (Unt.), KCl 0.6 M, sorbitol 1.2 M, or 37°C (heat) for 1 h, then washed and allowed to grow without the drug for 3 h. The graph represents the mean ± SD of >200 cells from 2 independent experiments. Statistical significance was calculated using a two-tailed unpaired Student’s t test. (C) Images of WT and rgf1Δ cells expressing LifeAct-GFP to visualize actin. Maximum-intensity projection of cells (n = 22) of the same size from each strain is included to show the distribution of actin patches along the cells. (D) Cells expressing rgf1-GFP and crn1-GFP (actin patches) or mCherry-atb2 (MTs) cultured separately, mixed, and then treated with DMSO, LatA 100 μm, or MBC 50 μm for 15 min. (E) LifeAct-mCherry (actin) and Rgf1-GFP localization in WT cells treated with KCl 0.6 M for 1 h and then washed and allowed to grow without stress for the indicated times. The localization of Rgf1-GFP in tea4Δ cells under the same conditions is shown in the lower panel. The maximum-intensity projection of 4 Z-slides (0.6 μm step-size) of fluorescence is shown. (F) Quantitation of the T-shaped cells in the WT, tea1Δ, gef1Δ, and tea1Δ gef1Δ cells grown to log phase in YES liquid medium at 28°C. The graph represents the mean ± SD of >500 cells from 2 independent experiments. Statistical significance was calculated using a two-tailed unpaired Student’s t test. (G) GFP fluorescence in WT and rgf1Δ log-phase cells expressing sty1-GFP. Cells either were untreated (upper panels) or treated with 1 mM H2O2 for 15 min (lower panels). The maximum-intensity projection of 6 Z-slides (0.5 μm step-size) of Sty1-GFP fluorescence is shown. (H) Extracts from wild type (WT-unt) and rgf1Δ cells (rg [file pbio.3002491.s007.pdf]

Figure 3

B

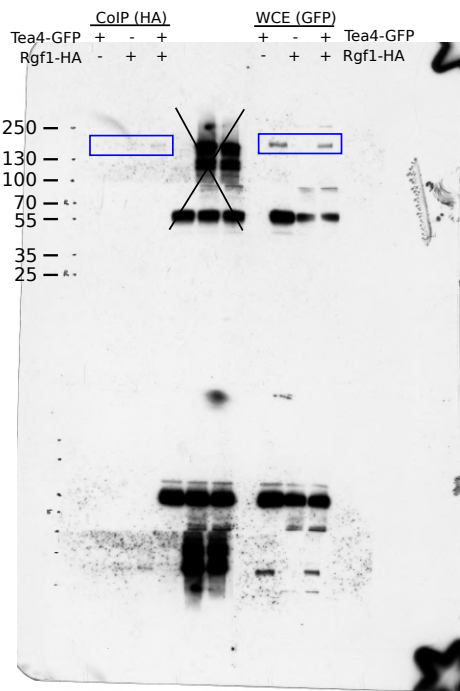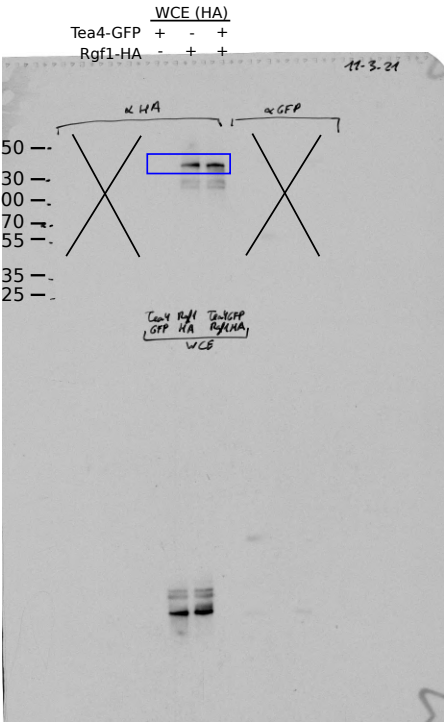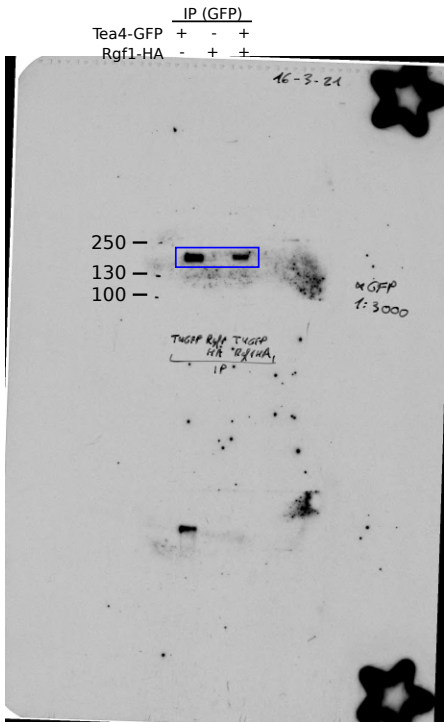

C

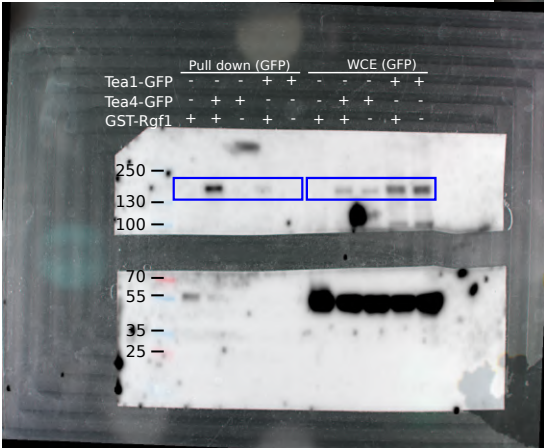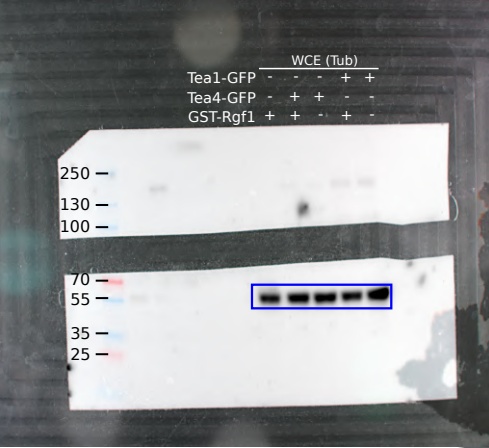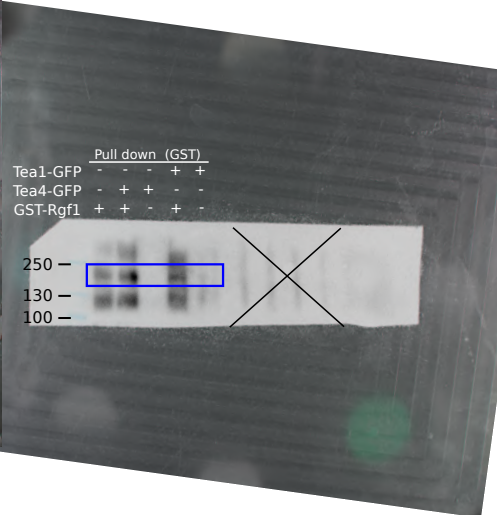

E

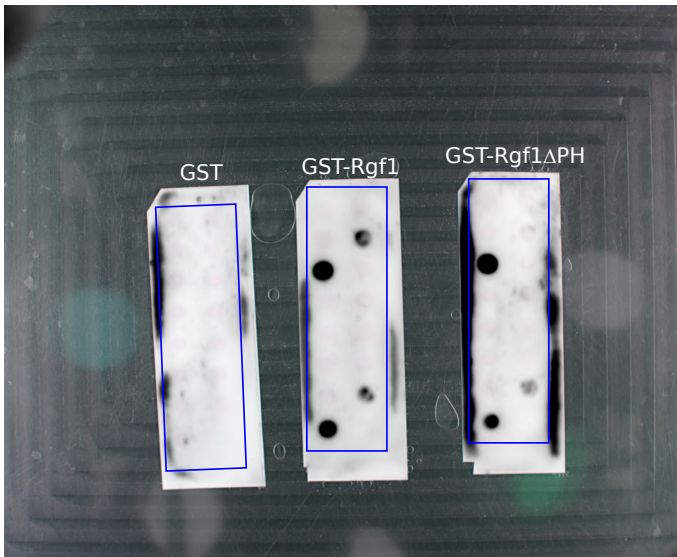

F

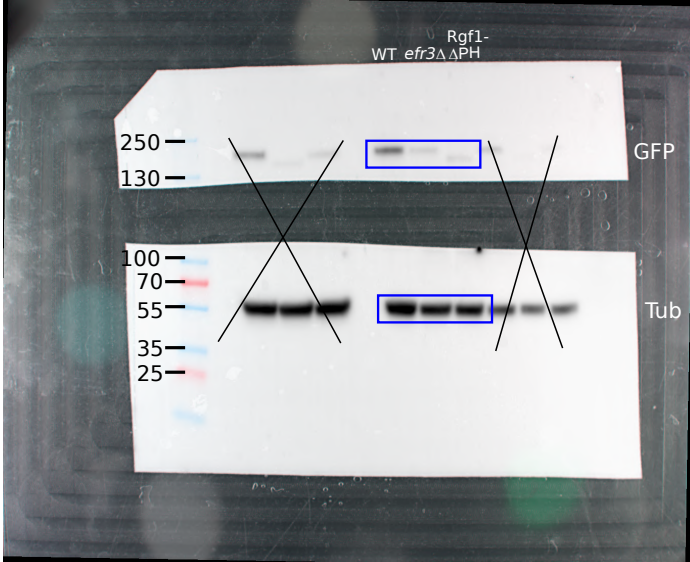

Figure 4

B

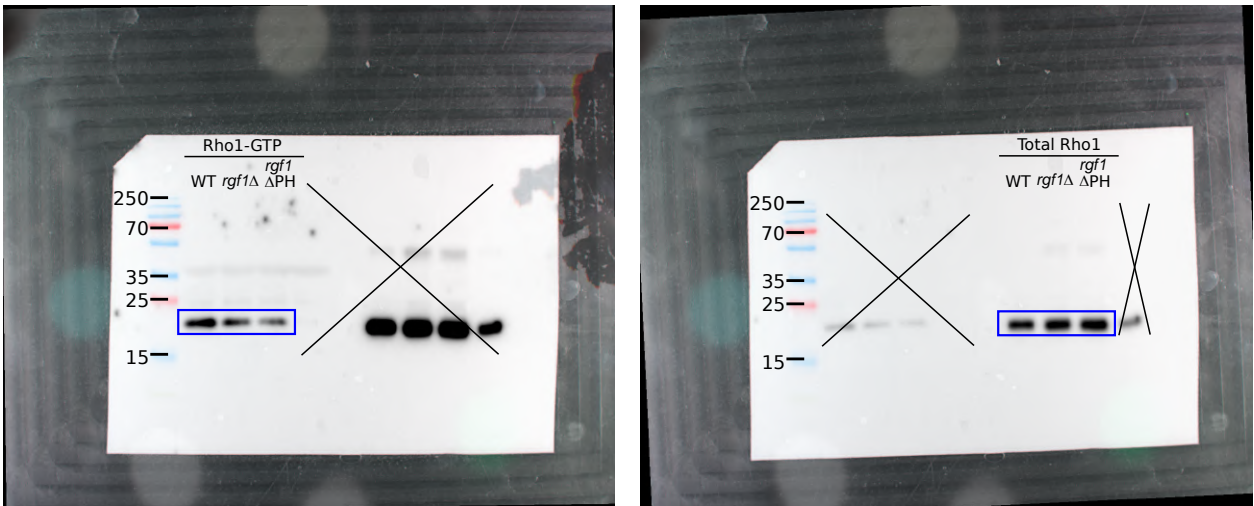

C

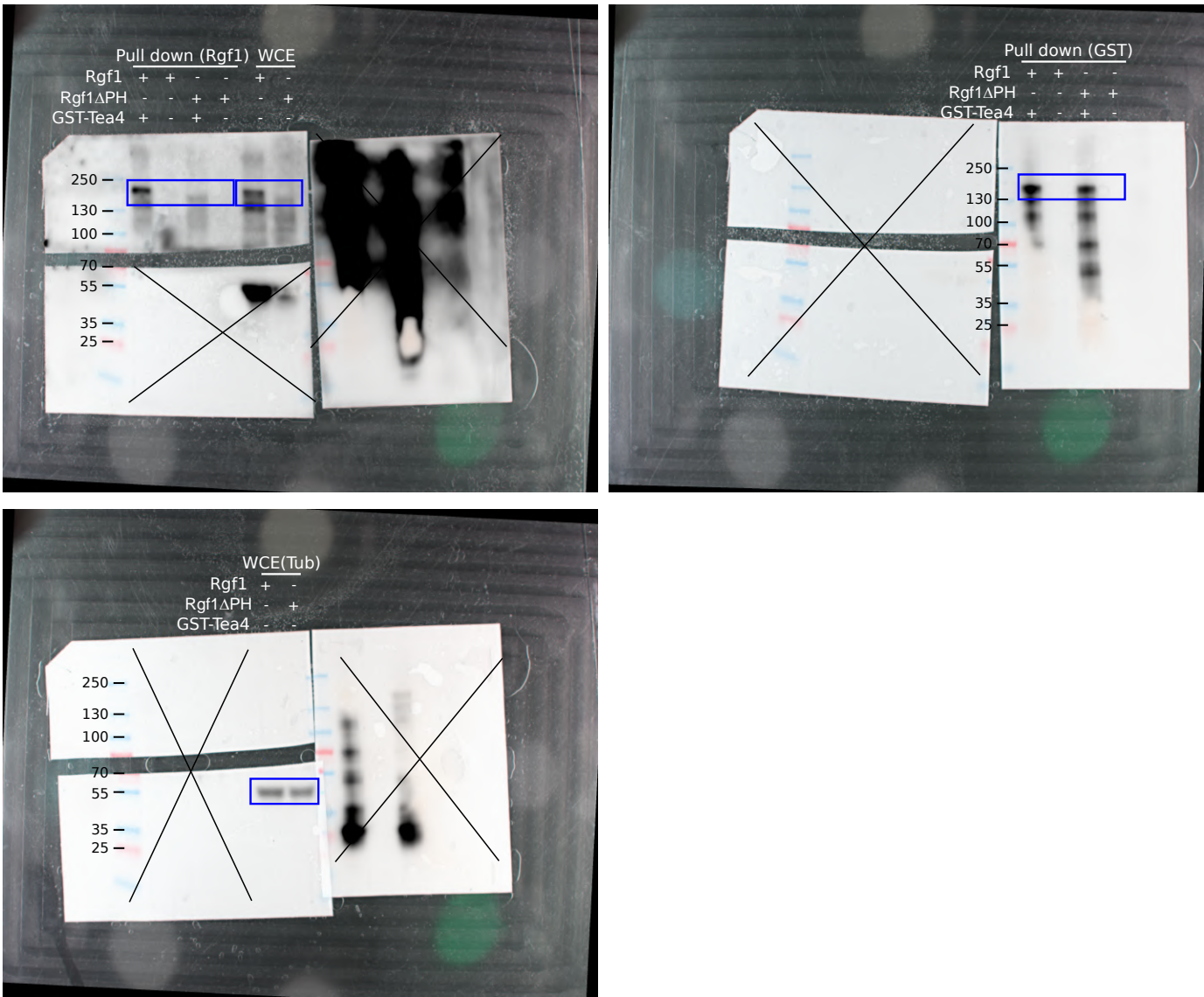

Figure 4

D

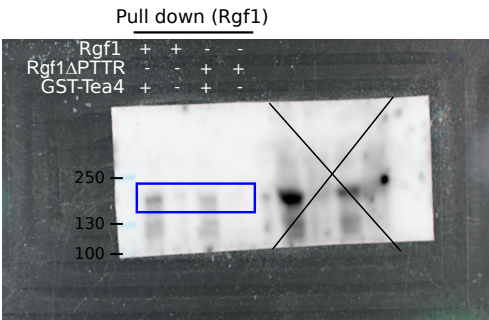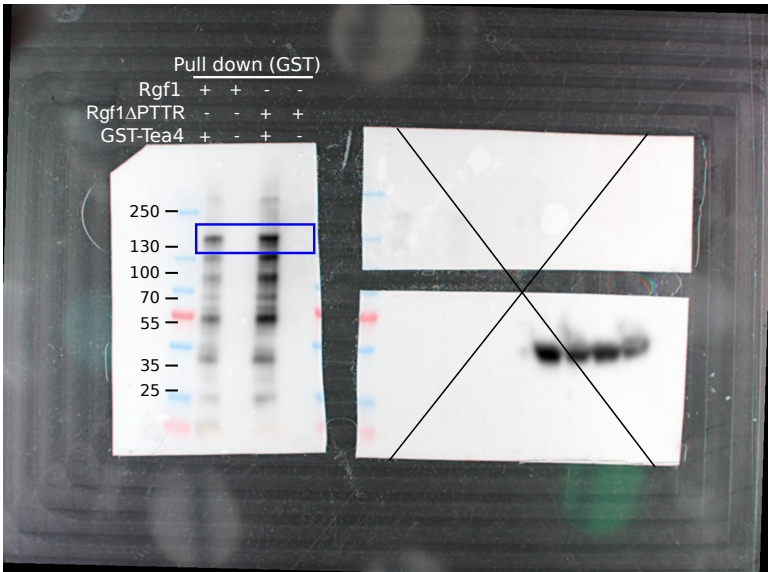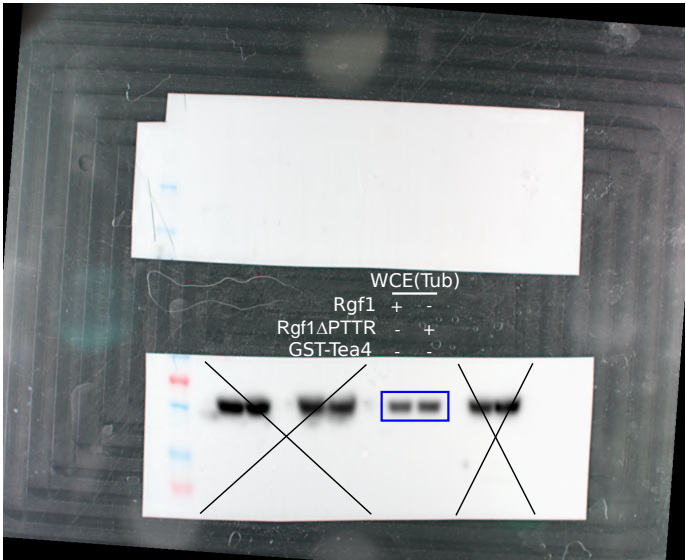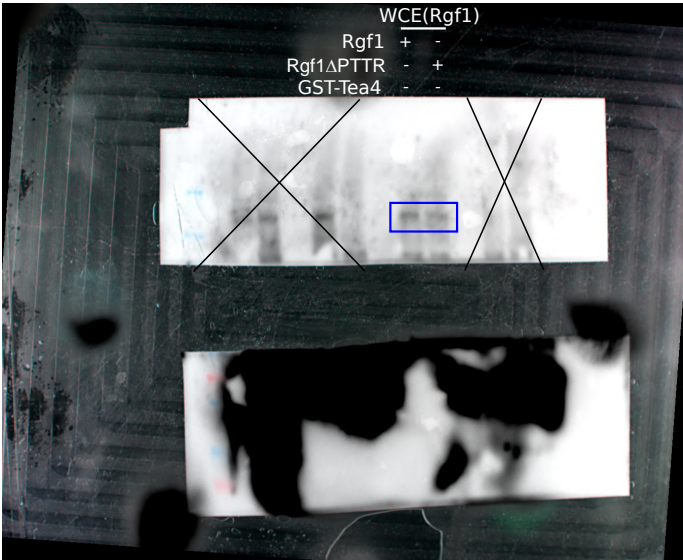

Figure S1

F

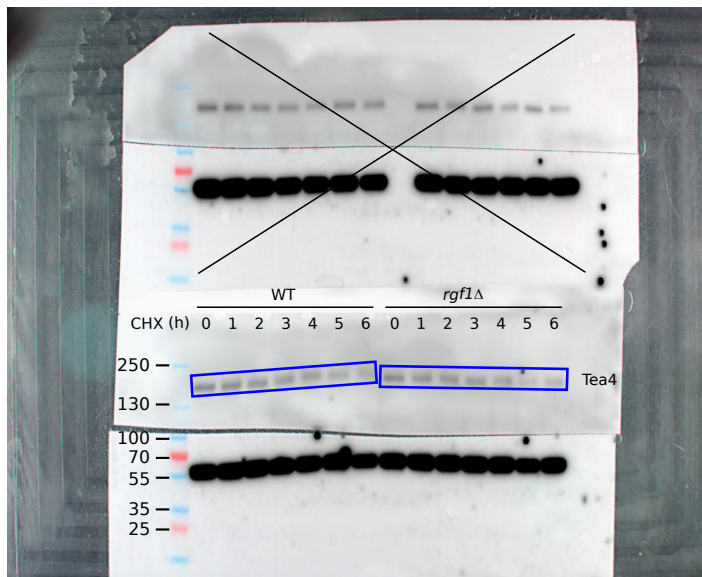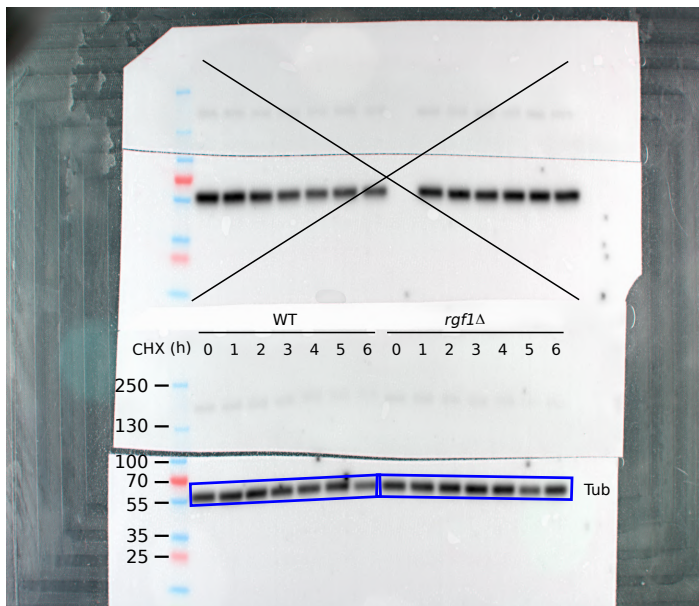

Figure S3

B

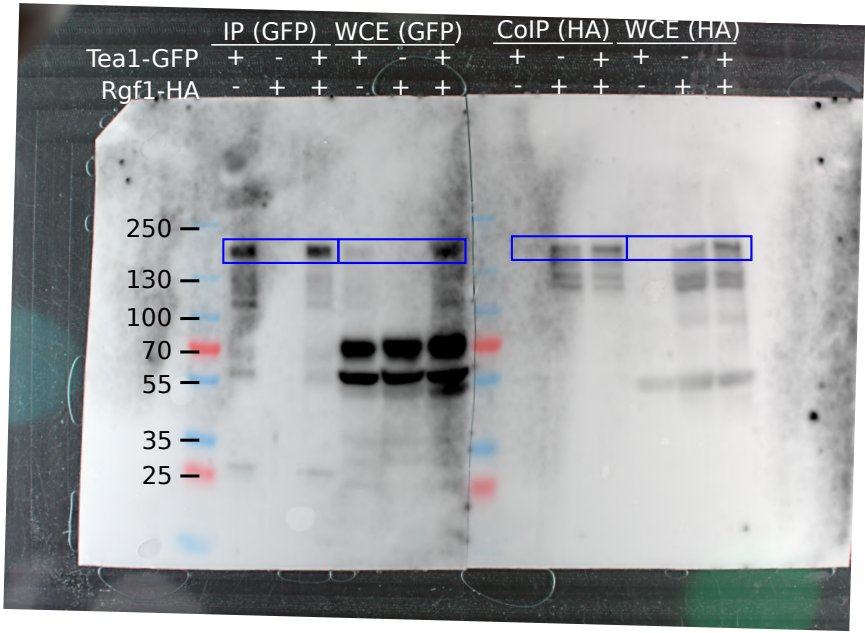

Figure S4

B

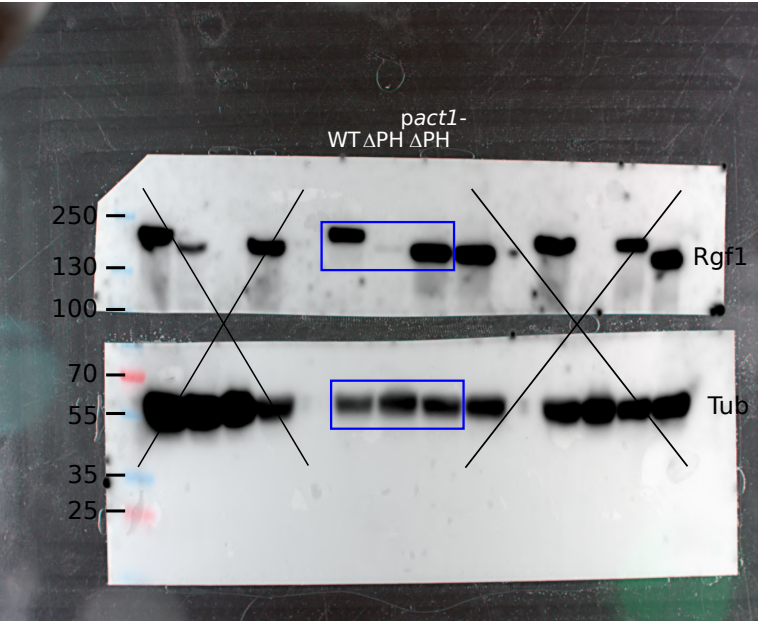

C

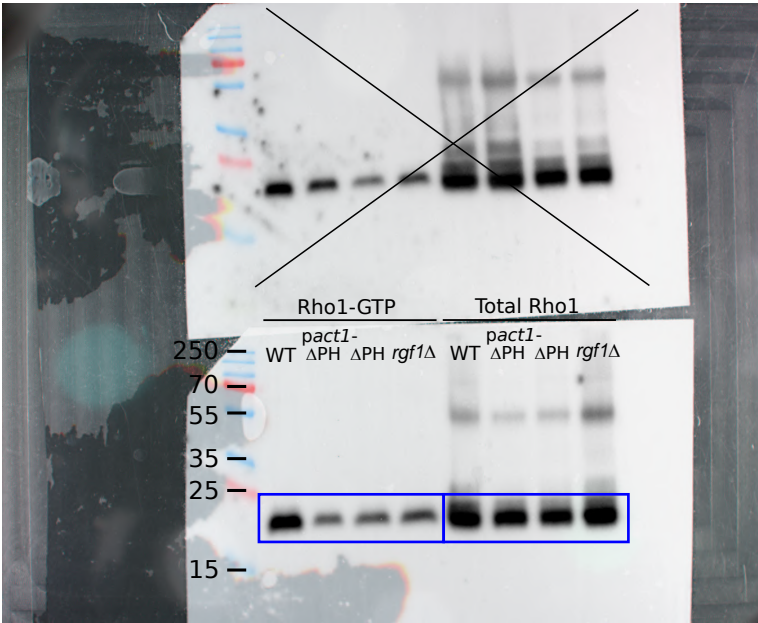

Figure S5

H

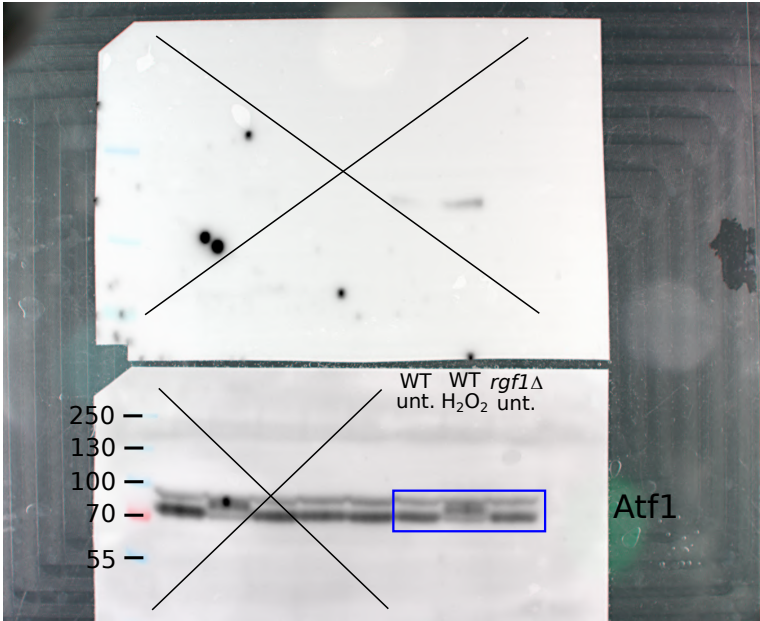

Supplement: S1 Raw Images — (PDF) [file pbio.3002491.s013.pdf]
