# Supplementary material for: Rho1 and Rgf1 establish a new actin-dependent signal to determine growth poles in yeast independently of microtubules and the Tea1–Tea4 complex
Source: PLoS Biol. 2024 Nov 7;22(11):e3002491. doi: 10.1371/journal.pbio.3002491 (PMC11602027; doi:10.1371/journal.pbio.3002491)
Supplement: Attachment — Submitted filename: Response to reviewers.pdf [file pbio.3002491.s015.pdf]

PBIOLOGY-D-23-03415R1

Dear Academic Editor/ Dr. Álvarez-García:

This is a revised version of the manuscript PBIOLOGY-D-23-03415R1, entitled "Rgf1 GEF activity toward Rho1 defines a new actin-dependent signal to determine growth sites independently of microtubules and Tea1"

Reviewers' comments:

Rev. 1:

In this manuscript, García et al. analyze the biological relevance of the Rho1 GEF Rgf1 in the establishment of polar growth in fission yeast. The work presents two main conclusions: 1) Rgf1 contributes to Tea4 and Tea1 polarity landmarks anchoring at the cell poles; 2) Two pathways define the growth poles in fission yeast: one dependent on microtubules and Tea1-Tea4 and another dependent on actin and Rgf1-Rho1.

Evidence in multiple organisms supports that precise cell polarization relies on interactions between the microtubule and actin cytoskeletons. In fission yeast, the precise location of polar growth activation is established by depositing the Tea1-Tea4 complex at the cell poles. Without this complex, cells cannot accurately position growth at the cell poles, leading to irregularly shaped cells. Additionally, when growth is reinitiated de novo, it might occur at a site other than the cell tips, resulting in the formation of T-shaped cells (as shown in seminal papers from Nurse, Sawin and Chang laboratories). Polarity establishment depends on Tea1 anchored at the poles by Mod5 and a small amount deposited at the cell ends in association with microtubules ends (Snaith and Sawin, 2003). Simultaneous disruption of both microtubules and actin cables still permits polarized cell growth, although the cells do not correctly position the growth poles (Bendezú and Martin, 2010). Indeed, de novo polarization after starvation depends on the polarized recruitment of sterol-rich domains, which relies on Tea1 and F-actin (Makushok et al., 2016).

The authors of this manuscript build upon these previous observations, some of which were not referenced, to demonstrate that Rgf1-Rho1 play a role in establishing polarity under specific circumstances. The data that leads to and extends point 1 are compelling but not entirely novel. In 2017, Dogdson et al. reported that deleting Rgf1 resulted in a consistent reduction in the fluorescence enrichment of polarity landmarks Tea1, Tea4 and Pom1 at the cell tips (http://dx.doi.org/10.1101/116749). However, this article has not been cited by the authors and is not mentioned in the introduction, despite its relevance to the manuscript's topic. The analysis that leads to point 2 is not that persuasive. It is important to note that while understanding the control of cell polarity is of great interest in the field of morphogenesis, it remains unclear whether the conceptual advances presented in this work are sufficient, given the uncertainty regarding Rgf1's target/s in the control of actin cytoskeleton, and the already previous published data that acknowledge the contribution of both microtubule-dependent signals and actin-dependent signals for the establishment of polarity. In summary, given the lack of mechanistic insight in the latter sections of the paper and the modest advancement in understanding presented in the former, coupled with the numerous reservations regarding data presentation,

experimental design, and quantification, I am not able to express great enthusiasm for the paper in its current state. Please refer to the comments below for further specifics regarding the manuscript.

1. Rgf1 is required to locate the Tea1-Tea4 complex properly and to integrate Tea4 in big clusters at the cell tip (Figure 1). As mentioned earlier, reduced localization of Tea1 and Tea4 in rgf1-null cells has already been described. The data are not novel, and previous work has not been appropriately cited.

Answer:

We find the results of Dodgson *et al*. (http://dx.doi.org/10.1101/116749) very interesting and have been following this preprint for a few years. However, because it has not been peer-reviewed, we initially decided not to mention it. Following your suggestion, it has been included in the revised version to provide a comprehensive context for our study.

Page 5 and line 117:

"Accordingly, a reduction in Tea1 and Tea4 localization in *rgf1Δ* cells has been described previously in a wide protein localization dependency study (42)."

We have also cited other two publications that the reviewer suggested (Bendezú and Martin, 2011) and (Makushok et al., 2016) in the Introduction.

Page 3, line 73:

Makushok et al., 2016: "Tea1 and Tea4 colocalize at the cell tips within sterol-rich membrane domains (18) to form clusters or nodes, (19)…."

Page 3, line 77:

Bendezú and Martin, 2011: "Nevertheless, in the absence of both microtubules and actin cables fission yeast still polarize growth, although less efficiently (21).

Figure 1A, B. Authors quantify fluorescence intensity of Tea4-GFP at the cell tips in WT vs rgf1- cells and claim that in rgf1- cells, the signal was visibly diminished. Is the signal reduced, or is it just that in most wild-type cells, Tea4-GFP is localized at the two poles and in rgf1- just in one pole? In panel B, the fluorescence intensity of Tea4-GFP in wild-type cells appears to be similar to that observed in the non-growing pole in rgf1- cells. Can the authors include measurements of wild-type cells to compare fluorescence intensity with the non-growing pole of rgf1- cells? Additionally, it would be interesting to know if other monopolar mutants, distinct from polarisome components, also show reduced Tea4 localization at the growing pole.

Answer:

Thank you for your suggestion. We have included measurements of Tea4-GFP fluorescence intensity in wild type and *rgf1Δ* cells at both the growing and non-growing poles in Figure 1B. The results show that fluorescence levels significantly decrease in the mutant at both poles. This indicates that the observed defect is not solely due to Tea4 being localized at both poles in the wild type and only at one pole in the *rgf1Δ* mutant.

Additionally, we have quantified Tea4 levels at the poles of a monopolar mutant unrelated to the polarisome, specifically the temperature-sensitive mutant *cdc10-129*, which exhibits a high percentage of monopolar cells at 37°C. As shown in the graph in Figure S1A, the fluorescence

levels at both poles in this mutant remain unchanged compared to the wild type. This result demonstrates that the localization defect observed in the *rgf1*Δ mutant is not due to its monopolar growth.

Fig 1B

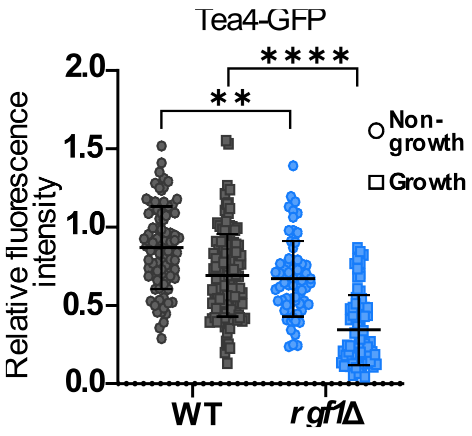

Fig S1A

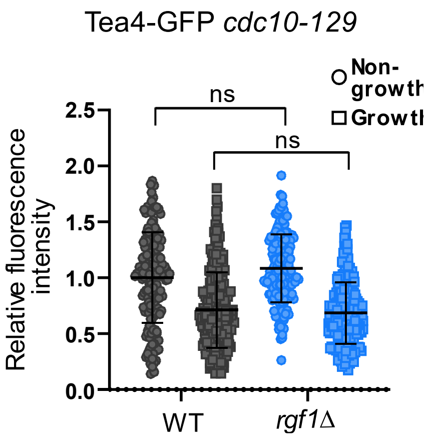

In the text (page 5 and line 126).

The sentence "We observed that 61% of the *rgf1*Δ cells accumulated Tea4 at the non-growing end (revealed by calcofluor staining)", has been replaced by "We observed that both wild-type and *rgf1*Δ cells accumulated Tea4 more at the non-growing end (revealed by calcofluor staining). However, compared to wild-type cells, the average fluorescence intensity of Tea4 in *rgf1*Δ cells was significantly reduced at both ends, although the decrease was greater at the growing pole (Fig. 1B). This reduction is characteristic of *rgf1*Δ cells. In contrast, when performing the same experiment in *cdc10-129* cells, which exhibit a high percentage of monopolar cells at 37°C (~90%), we observed no appreciable differences in Tea4 fluorescence intensity at either end compared to wild-type cells (Fig. S1A)".

In addition, the legend of Figure 1B and Figure S1C have been modified accordingly.

Page 6, line 146.

"The graphic represents the mean ± SD of the relative fluorescence intensity of Tea4-GFP measured at the growing end and the non-growing end of the WT and *rgf1*Δ (n >70) cells' ".

Page 40, line 1010.

Figure S1. "(A) The graphic represents the mean ± SD of the relative fluorescence intensity of Tea4-GFP measured at the growing and non-growing ends of WT cells (n > 150) and *cdc10-129* cells (n > 150) grown for 4 hours at 37°C. Calcofluor staining was used to differentiate the growing poles from the non-growing poles."

Figure 1C. The authors claim that there is a wide co-localization between Tea1 and Tea4 in the absence of Rgf1 and conclude that both proteins displayed similar localization defects. Nevertheless, many red spots corresponding to Tea1-tdT in the merged image can be observed, suggesting that the effect on Tea1 localization is not severe. It is difficult to conclude from the images provided that Tea1-tdT is mainly localized to one pole. A more careful analysis of Tea1 localization in rgf1-null cells, including wild-type cells as a control, is necessary. Additionally, the

authors state that Tea1 is mainly localized to the non-growing end (lines 164), but this information is not shown in panel C or panel B.

Answer:

We have updated panel Figure 1C to include the colocalization of Tea1 and Tea4 in both the *rgf1Δ* mutant and the wild type. These new images provide a clearer demonstration that nearly all Tea1 spots coincide with Tea4 spots. However, it is important to note that Tea1 is more abundant compared to Tea4.

Minor differences in the localization between Tea1 and Tea4 were observed in both strains, likely due to the time lag between capturing images in the green and red channels. Additionally, a more detailed analysis of the two cells shown in Figure 1C has been included in Figure S1C. This was done by performing a line scan from pole to pole, covering the entire width of the cell. The results show that the majority of the fluorescence peaks detected in the green channel are also observed in the red channel, indicating a high degree of colocalization of both proteins in both the wild type and the mutant strain.

Fig 1C

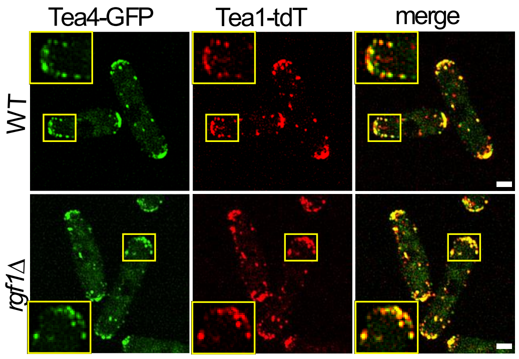

Fig S1C

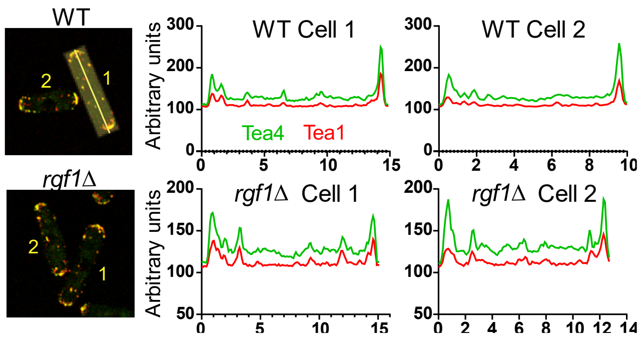

As the reviewer suggested, we have analyzed the localization of Tea1 more carefully. To this end, we have included photographs in Figure S1D showing the localization of Tea1-GFP in the wild type and the *rgf1Δ* mutant. Additionally, we quantified the fluorescence intensity of Tea1-GFP at the poles of both strains comparing the fluorescence intensity of the growing and non-growing poles. In both cases, the fluorescence intensity of Tea1 is lower in the mutant than in the wild type. Similar to our observations for Tea4, in the *rgf1Δ* mutant, the pole with the least Tea1 fluorescence is the growing pole.

Fig S1D

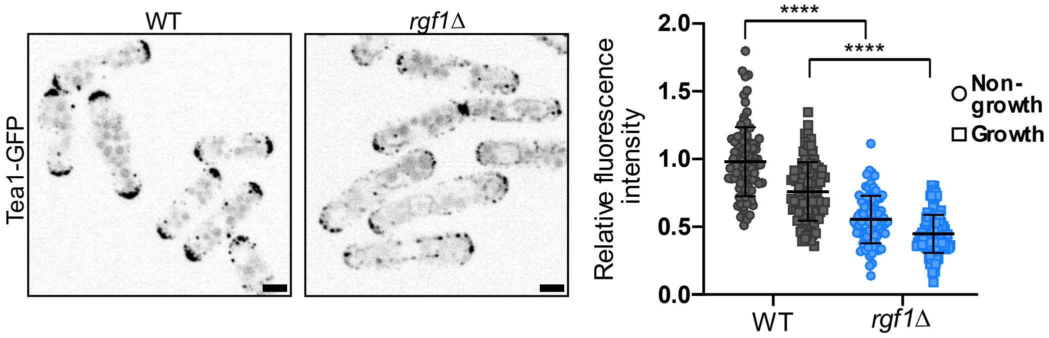

In the text (page 7, line 171) of the revised manuscript, we have replaced Fig. 1C for the new Fig. 1C, that includes the colocalization of Tea1 and Tea4 in both the *rgf1Δ* mutant and the wild type. In addition, we have included in Fig. S1C a line scan from pole to pole of cells of Fig. 1C showing

a detailed colocalization of both proteins, and in Fig. S1D pictures of Tea1 localization at both strains and the corresponding quantification.

In the text, page 7 and line 173, we have included Fig S1D

"Given that in *rgf1*Δ cells, Tea1 also localized mainly to the non-growing end (Fig S1D), our results suggest that the Tea1 mislocalization could be a consequence of Tea4 mislocalization".

The legend of Figure 1C and S1C and D have been modified accordingly.

Page 6, line 148. Fig 1(C) "Representative images of Tea4-GFP (green) and Tea1-tdTomato (red) localization in the WT and *rgf1*Δ cells. The maximum-intensity projection of six deconvolved Z-slides (0.5 µm step-size) is shown".

Page 40, line 1015. Figure S1. "(C) Maximum projection images of the cells from Figure 1C show the merged localization of Tea4-GFP (green) and Tea1-tomato (red) in wild type (WT) and *rgf1*Δ cells (left panels). The fluorescence intensity profiles for green and red channels along a line across the poles, covering the cell width, are shown for cells 1 and 2 of WT and *rgf1*Δ cells, respectively, in the right panels. Scale bar 2µm."

Page 40, line 1019. Figure S1. "(D) GFP fluorescence in cells expressing *tea1-GFP rgf1*$^{+}$ and *tea1-GFP rgf1*Δ grown to log-phase at 28°C in YES medium. The maximum-intensity projection of six Z-slides (0.5 µm step-size) of Tea1-GFP fluorescence is shown. The graphic represents the mean ± SD of the relative fluorescence intensity of Tea1-GFP measured at the growing and non-growing ends of WT (n > 80 for each end) and *rgf1*Δ (n > 80 for each end) cells."

Figure 1H. The authors suggest that the limited amount of Tea4-Tea1 at the growing pole underlies the curly phenotype of microtubules. Alternatively, it is possible that the curly microtubules and the extended dwelling time at the tips of rfg1-null cells contribute to the reduced amount of Tea1-Tea4 at the poles. In this context, it has been reported that Amo1 cooperates with Tea1 in maintaining cell polarity by regulating the proper dynamics of microtubules. Consequently, when Amo1 is absent, dwelling time at the tips is also lengthened, leading to curly microtubules and irregular Tea1 deposition (Pardo and Nurse, 2005). In addition, the double mutant amo1-null tea1-null forms branches during exponential growth, a phenotype not observed in the single mutants. These phenotypes are similar to those observed in the absence of Rgf1; therefore, microtubules may play a partial role or Rgf1 might be regulating Amo1.

Answer:

We cannot rule out the possibility that in the *rgf1*Δ cells the curly microtubules and the extended dwelling time at the tips contribute to the reduced amount of Tea1-Tea4 at the poles. However, for several reasons, we believe that this is not the case. First, in the *rgf1*Δ mutant, we observed one curly microtubule in only 10% of the cells after 4 hours at 37°C. In contrast, the percentage of cells with one curly microtubule at 28°C is marginal (<1%), which is the temperature at which we collected data for most of the experiments showing mislocalization of Tea4 at the poles. Second, our results suggest that Rgf1 is not directly involved in interphase microtubule organization. Similar to the wild type, *rgf1*Δ cells show 2-5 microtubule bundles during interphase and the microtubule polymerization and depolymerization rates in the wild type and *rgf1*Δ cells are alike (Fig S1G).

Regarding the similarity of the curly microtubules and a lengthened dwelling time at the tips in *amo1*Δ and *rgf1*Δ cells, the bases of these phenotypes may be different. Cells lacking Amo1 show abnormal MT behavior and consequently problems to maintain the nucleus in the middle of the

cell. In addition, Tea1 accumulates at the lateral cortex in *amo1*Δ. However, *rgf1*Δ cells do not show any of these defects, suggesting that both proteins act in different processes. Other differences with *amo1*Δ cells concern their sensitivity to MBC and TBZ depolymerizing agents. Amo1 null mutants are resistant to MBC and their microtubules are more stable than in wild-type cells (Pardo and Nurse, 2005). In contrast, the *rgf1*Δ cells are hypersensitive to MBC (our unpublished observations). We have shown that Rgf1-GFP lacking the DEP domain localizes to both SPBs and the spindle (Muñoz et al., 2014). Thus, in this case, it is possible that the sensitivity to MBC arises from spindle formation and chromosome segregation defects, as has been shown for other *S. pombe* mutants.

Finally, the elimination of Tea1 in the *rgf1*Δ and *amo1*Δ backgrounds also yields differences. While only 10% of the exponentially growing *tea1*Δ *amo1*Δ cells form branches, more than 45% of the *tea1*Δ *rgf1*Δ cells are branched. We have obtained double mutants *rgf1*Δ *amo1*Δ. Neither in log-phase (there are no branched cells), nor in stationary phase refeeding experiments did we observe a higher number of branched cells in the double mutant compared to the individual *rgf1*Δ nor *amo1*Δ mutants (Fig 1, not included in the revised version). This suggests that Rgf1 and Amo1 do not cooperate in the maintenance of cell polarity, or that Rgf1 might be regulating Amo1, which will require further investigation.

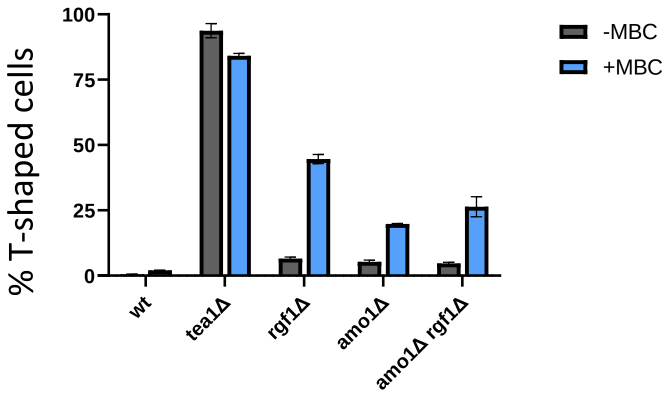

Fig 1. The percentage of cells forming branches in the indicated strains. The cells were grown to the stationary phase for 3 days, and then were treated with DMSO (-MBC) or MBC (+MBC; 50 μg/ml). The mean ± SD of > 200 cells from three independent experiments is shown.

Finally, it has been shown that an increase in the pool of free monomeric actin is sufficient to cause a switch from monopolar to bipolar actin in fission yeast cells, provided that both cell ends are marked as potential sites of growth (Rupes et al., 1999). Many monopolar mutants can induce NETO after this treatment, and therefore able to recognize both cell ends and to use them as sites for growth. It will be interesting to know whether this is the case for Rgf1.

Answer:

Thank you for this suggestion. We have done this experiment previously using immunofluorescence with Rhodamine/Phalloidin and have now repeated it with cells tagged with Life-act (Fig 2, not included).

In our setup, both *cdc10-129* and *cdc10-129 rgf1Δ* cells blocked for 4 hours at 37°C (before LatA treatment) showed a monopolar actin distribution (Fig 2, upper panel). After a 5-minute LatA pulse, wild-type cells frequently switched to a bipolar actin distribution (~90%), whereas only ~60% of *cdc10-129 rgf1*Δ cells showed bipolar actin distribution (Fig. 2, lower panels and graph). This result suggests that the NETO defect characteristic of the *rgf1*Δ mutant could be at least partially due to problems in recognizing the new end. In Figure 2 the monopolar cells (*) have actin at both poles, with actin being less concentrated at the non-growing end. To distinguish

between the ends we have double-stained these cells with Calcofluor which marks the growing end.

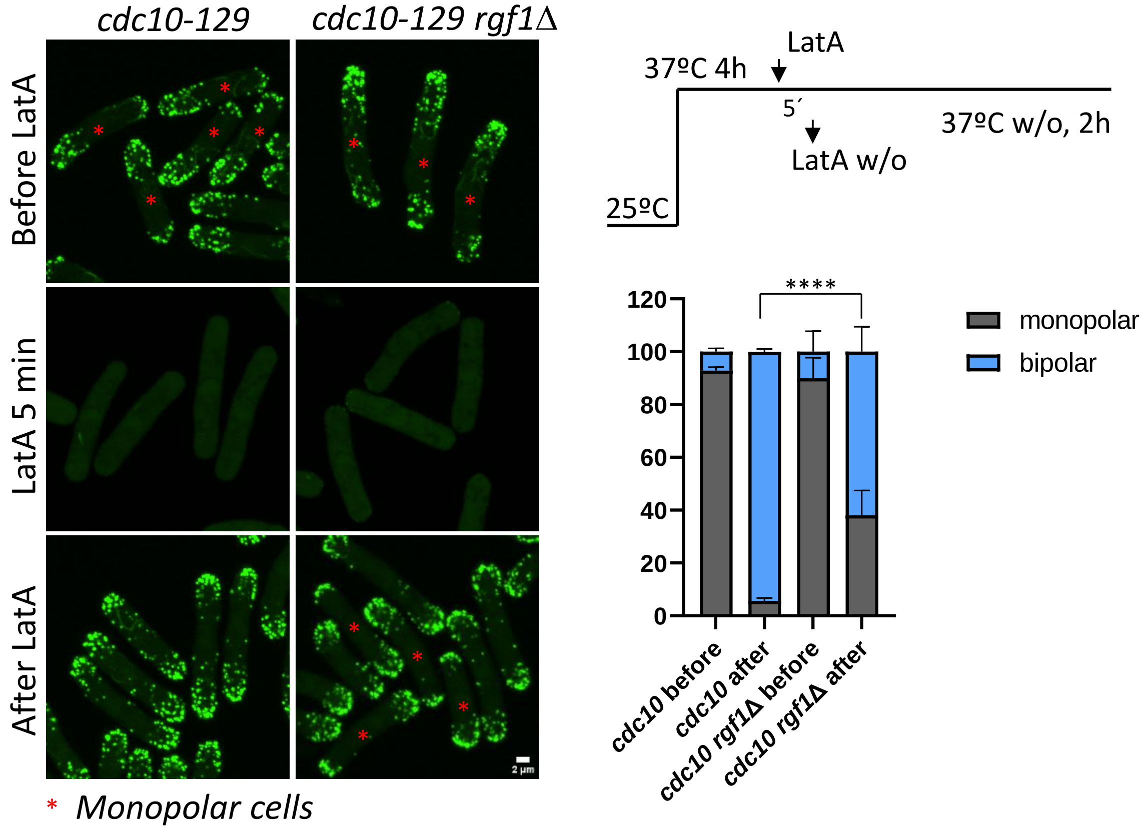

Fig2. Schematic representation of a LatA pulse experiment. Micrographs of cells before (top panels), during (middle panels) and 2h after LatA pulse (lower panels) are shown. Bar,2µm. The graph indicates the number of monopolar and bipolar cells in *cdc10-129* and *cdc10-129 rgf1*Δ cells before and after the LatA pulse. The mean ± SD of > 150 cells from two independent experiments is shown.

2. Rgf1 cooperates with Mod5 in Tea4, anchoring to the cellular poles (Figure 2). According to the literature, in mod5-null cells, Tea1 remains associated with microtubule ends, but it no longer accumulates at both cell tips. The authors claim that rgf1-null cells show a similar defect (line 212), however, it remains to be precisely determined the impact of rgf1 deletion on Tea1 localization. From the data provided in Figure 2A, this can only be concluded for Tea4, and, in this case, signal can be appreciated at one of the poles. Authors should analyze the impact of Rgf1 and Mod5 deletion on Tea1 localization and include quantification of fluorescence enrichment at the cell tips for these experiments. Only then can they conclude that Rgf1 and Mod5 collaborate to position the polarity markers at the cell tips (lines 251-252).

Answer:

As suggested by the reviewer, we analyzed the localization of Tea1 in *rgf1* and *mod5* deletion mutants and quantified the fluorescence enrichment at the cell tips for these experiments (Fig. S2A). Similar defects were observed of both Tea1 and Tea4 proteins in *mod5Δrgf1Δ* cells. Additionally, we have included the quantification of Tea4 fluorescence in Fig.2A.

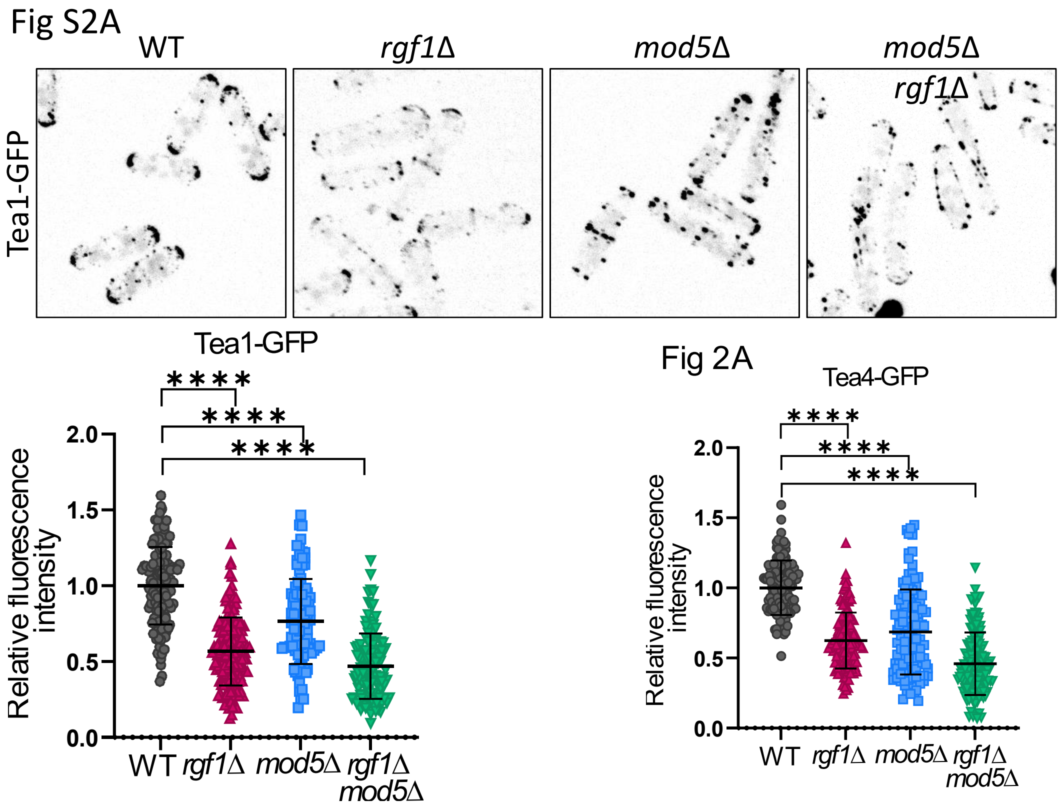

The text, page 9, line 222, the manuscript has been modified as follows…

"we analyzed the localization of Tea4 and Tea1 in the double mutant *rgf1Δmod5Δ* compared with the single mutant".

Page 9, line 227. "The Tea1 signal at the poles exhibited a pattern similar to that of Tea4 in both single and double mutants (Fig S2A)", suggesting that Mod5 and Rgf1 share a function in Tea1–Tea4 anchoring**.**

We have included a new panel in Fig 2A and changed the corresponding Fig 2A legend.

Page 10, line 234. Figure 2. "The graphic represents the mean ± standard deviation (SD) of the relative fluorescence intensity of Tea4-GFP measured at the cell tips in the wild type (WT), *rgf1Δ*, *mod5Δ* and *rgf1Δ mod5Δ* cells (n> 120). WT levels were used for normalization. Statistical significance was calculated using a two-tailed unpaired Student's t test."

We have included a new Fig. S2A and the corresponding Fig. S2A legend.

Page 41, line 1035. Figure S2. "(A) Representative images of the indicated strains producing Tea1-GFP. The maximum-intensity projection of six Z-slides (0.5 µm step-size) is shown. Scale bar: 2 µm. The graph represents the mean ± SD of the relative fluorescence intensity of Tea1-GFP measured at the cell tips in the wild type, *rgf1Δ*, *mod5Δ* and *rgf1Δ mod5Δ* cells (n > 100). WT levels were used for normalization."

Figure 2B presents an inconsistency between the results reported in this manuscript and those in Snaith and Nurse (2003). In the latter study, the percentage of branched cells in polarity re-establishment experiments in the presence of MBC (50 g ml-1) in mod5- cells reaches 84%, which is not statistically significantly different from tea1-null cells. In this manuscript, however, the authors indicate that only the rgf1-mod5- double mutant phenocopies tea1-null cells in the presence of MBC. The authors should clarify this discrepancy and include a statistical analysis of the results in Figure 2B. Finally, it is recommended that the authors include TEA4-null cells in polarity re-establishment experiments and generate the double cdc11 tea4- mutant to determine the percentage of T-shaped cells in Figure 2B, C. This will provide a more comprehensive understanding of the phenotype of the rgf1- mod5- double mutant.

Answer:

Regarding the last question, we have repeated the refeeding experiments, this time including *tea4*Δ cells (Fig. 2B and S2B), and have included a statistical analysis of the results in Fig. 2A. In addition, *tea4*Δ *cdc11* double mutant analysis is now shown in Fig. 2C and S2C.

Our values may be slightly different from those of Snaith and Sawin (2003). We used minimal medium (MM) instead of rich medium. Additionally, while they grew cells in stationary phase at 25°C before diluting them into fresh medium at 32°C (which introduces additional stress), we maintained a constant temperature of 28°C throughout the experiment. The reason for using MM medium is that when *rgf1*Δ cells are returned to fresh medium after stationary phase, a significant percentage of cells lyse, likely due to defects in the cell wall, and this percentage is reduced in MM.

According to the reviewer's suggestion and our results, we have reorganized the paragraph on page 10, line 254. It was never our intention to imply that the *rgf1*Δ*mod5*Δ double mutant is the only that phenocopies *tea1*Δ in the presence of MBC, especially since *mod5*Δ cells also show a high percentage of branches, similar to the double mutant in the presence of MBC. In the revised version, we have clarified that only *rgf1*Δ*mod5*Δ cells, in the presence of MT (-MBC), behaved similarly to the *tea* mutants, reaching over 50% of T-shaped cells. This observation is key to proposing the additive effect of both Mod5 and Rgf1 in the establishment of polarity after refeeding.

The text of the revised version has been modified accordingly, page 10, and line 254.

"Consistent with the "poor" localization of Tea4 in the cell cortex, the *rgf1*Δ and *mod5*Δ cells showed polarity defects when treated with MBC (52% and 73% in *rgf1*Δ and *mod5*Δ cells, respectively) and marginal defects in the absence of MBC. In contrast, ~83% of the *tea1*Δ and 63% of the *tea4*Δ cells displayed the characteristic T-shaped pattern, with or without MBC treatment (Fig 2B and S2B) (16). Interestingly, the *rgf1*Δ*mod5*Δ cells behaved similarly to the *tea* mutants, reaching >50% T-shaped in the presence of MT (-MBC) (Fig 2B and S2B)".

We also have included a more detailed description of the procedure used in the refeeding experiments is provided in the Methods section. Page 27, line 682: "For refeeding experiments, we have incubated the cells at 28°C for 3 days in liquid EMM, to reach stationary phase, and diluted them 1:20 in fresh medium to let them grow for 3 hours at 28°C."

Fig. 2 has been modified accordingly.

We have modified Figure 2B to include the *tea4*Δ mutant in the refeeding experiments. The figure legend has also been updated to specify that the experiments were conducted at 28°C and to include the statistical significance calculated using a two-way ANOVA test.

Fig 2B

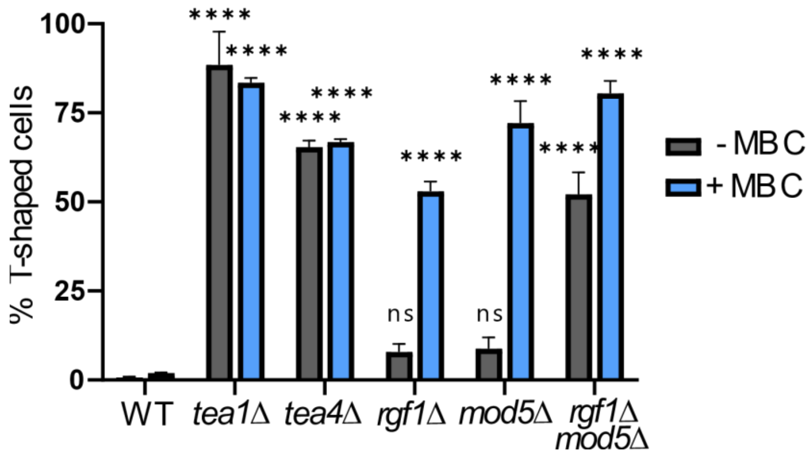

Figure 2C has been modified including *tea4Δ cdc11* double mutant without changes in the figure legend. The experiment was repeated at least 5 times.

Fig 2C % T-shaped cells

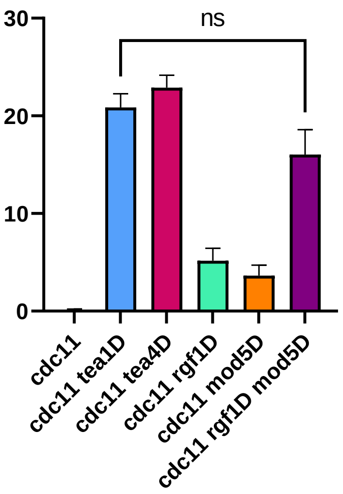

3. Rgf1 interacts with the cell-end marker Tea4 and binds to phosphatidylinositol-4-phosphate through its PH domain (Figure 3). From Figure S3, the authors conclude that Tea1 and Rgf1 do not interact. Nevertheless, it is recommended that the experiments be repeated under more stringent conditions, as an unspecific band appears that may mask a subtle interaction. This will ensure the validity of the findings and provide reliable insights into the relationship between Tea1 and Rgf1. In the pull-down assays a slight precipitation of Tea1 can be observed. Authors should demonstrate whether this is mediated by Tea4 binding.

Answer:

We have repeated the experiment multiple times under various stringent conditions. The figure below presents the results from a different experiment. Additionally, we attempted to establish the Tea1-Rgf1 interaction using other methods such as yeast two-hybrid assays (Figure 3D) and in vitro pull-down assays (Figure 3C), but these also yielded negative results.

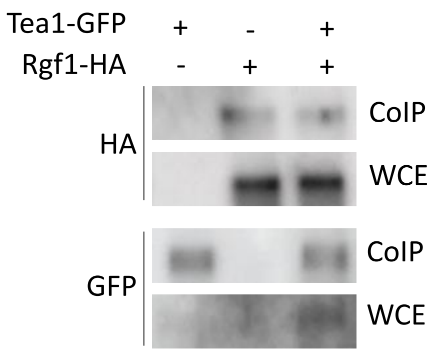

Fig. 3. Coprecipitation of Rgf1 and Tea1. Extracts from cells producing Tea1-GFP, Rgf1-HA, and Tea1-GFP Rgf1-HA were precipitated with GFP-trap beads and blotted with anti-HA or anti-GFP antibodies (co-immunoprecipitation and immunoprecipitation). Western blot was performed on total extracts to visualize total Tea1-GFP and Rgf1-HA levels (whole cell extracts).

In Figure 3F-H, the authors investigate the significance of the PH domain in the localization of Rgf1 at the poles. This analysis is not entirely novel, as previous research (Muñoz et al., 2014) characterized a version of Rgf1 lacking 56 amino acids in the PH domain and demonstrated that the normal localization of Rgf1 at the two tips was disrupted, with the signal primarily being monopolar. In that paper, the impact of the absence of the PH domain on the fluorescence intensity and protein level of Rgf1 was reported to be less severe than in the current study. The authors do not clarify the reasons for this discrepancy.

Answer:

Thank you for the suggestion. In our previous paper (Muñoz et al., 2014) we studied the Rgf1ΔPH mutant, which lacks 56 internal amino acids within the PH domain (residues 881–937). In this paper, we have created a more extensive deletion mutant (residues 722-1003), eliminating the entire PH domain. This more extensive deletion likely results in a more pronounced localization defect compared to the previously studied mutant.

In plasmid and DNA manipulations page 27 and line 699, we have included:

"For localization of the Rgf1 lacking the PH domain (pGR145), pGR41 (pJK148-*rgf1*-GFP) was mutagenize, followed by elimination of the entire PH domain between residues 772-1003)"

Furthermore, the role of phosphorylated phosphoinositides, specifically phosphatidylinositol 4,5-biphosphate (PIP2), in Rgf1 localization was demonstrated by Deng et al. (2005) in a JBC paper, which is not cited by the authors. In this work, the absence of PIP5K Its3 activity led to the severe disruption of Rgf1 localization at the cell tips.

In accordance with the reviewer suggestion, we have included Deng et al. 2005 citation and reorganized the following paragraph:

Page 14, line 343:

"It has been previously described that mutants in phosphatidylinositol kinases, which present an imbalance in the levels of PI(4)P and PI(4,5)P_2, show aberrant localization of Rgf1 in the PM (48,49). Moreover, cells lacking *efr3*$^{-}$, a PM scaffold for the PI(4)P kinase Stt4, show an important reduction of Rgf1 at the cell division site (49). We found that the localization of Rgf1 at the cell poles was compromised in the *efr3*Δ cells (Fig 3G). In addition, we detected…."

4. Tea4 accumulation at the cell ends depends on Rgf1 anchoring to the PM and Rho1 activation (Figure 4).

Figure 4A shows that in Rgf1-null, rgf1-PH, rgf1-PTTR mutants, Tea4-GFP is equally distributed in many cells in both poles, whereas previously (Figure 1), it was claimed to accumulate at the non-growing end. To provide a more accurate analysis of this phenotype, the authors should measure the relative fluorescence intensity for all strains depicted at the non-growing and at the growing poles. Additionally, there may be an additive effect of both the lack of PH and the absence of the four amino acids critical in the GEF domain.

Answer:

As the reviewer suggested, we have quantified the fluorescence intensity of Tea4-GFP in all strains at the growing and non-growing poles, which has been included in Figure S4A. As expected, higher fluorescence intensity was observed at the non-growing pole in all the analyzed strains. However, all mutants showed lower fluorescence levels than the wild type at both poles.

Additionally, we have included in the experiment the *rgf1*ΔPH-ΔPTTR double mutant, which lacks the 4 amino acids of the GEF domain and the PH domain of the *rgf1*$^+$ gene simultaneously, to analyze a possible additive effect of both mutations. As can be seen in the photographs and graphs included in Figures 4A, S4A and S4G this does not appear to be the case.

We have included a sentence in page 17 and line 418:

"The elimination of the PH domain and mutation of the catalytic site simultaneously has no additive effect, demonstrating this link (Fig 4A, S4A and S4G)."

We have modified Figure 4A, including *rgf1*ΔPH-ΔPTTR strain and the figure legend accordingly.

Fig 4A

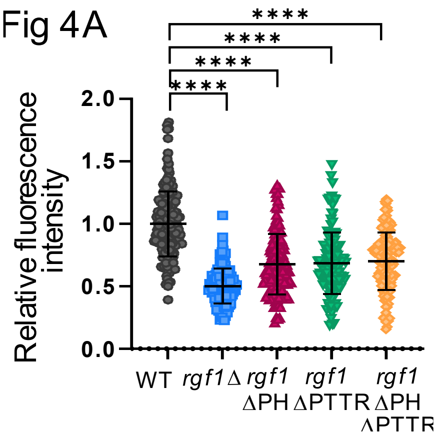

Page 15 and line 382:

Fig. 4. "(A) Representative images of Tea4-GFP localization in the WT, *rgf1*Δ, *rgf1*ΔPH, *rgf1*ΔPTTR, and *rgf1*ΔPH-ΔPTTR cells……. The graphic represents the mean ± SD of the relative fluorescence intensity of Tea4-GFP (n > 130) measured at the cellular tips. Statistical significance was calculated using a two-tailed unpaired Student's t test.

We have modified Fig. S4A, including the relative fluorescence intensity for all strains depicted at the non-growing and at the growing poles and we have included the *rgf1*ΔPH-ΔPTTR strain. The figure legend has been modified accordingly.

Fig S4A

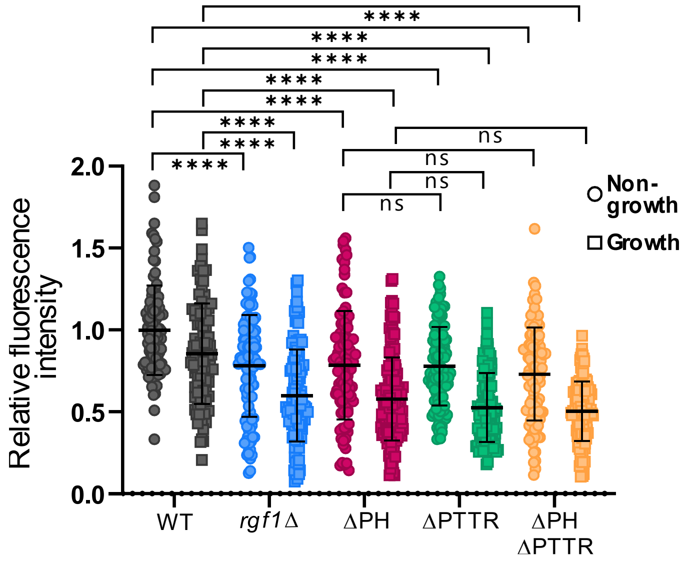

Pag 42, line 1053

Figure S4. "(A) The graphic represents the mean ± SD of the relative fluorescence intensity of Tea4-GFP measured at the growing and non-growing ends of the strains indicated (n > 100 for each end). Calcofluor staining was used to differentiate the growing poles from the non-growing poles."

We have modified Fig. S4G, including the percentage of T-shaped cells after refeeding for *rgf1*ΔPH-ΔPTTR strain, and the figure legend accordingly.

Pag 42, line 1069

Figure S4. "(G) The percentage of cells WT, tea1Δ, rgf1Δ, rgf1ΔPH, pact-rgf1ΔPH and rgf1ΔPH-ΔPTTR cells forming branches 3 hours after release to growth after 3 days in stationary phase, in the absence and in the presence of MBC (50 µg/ml). The mean ± SD of > 200 cells from three independent experiments is shown. Statistical significance of each strain compared to the wild type was calculated using a two-way ANOVA test. ****P< 0.0001; *P < 0.05; ns = non-significant."

Figure 4B indicates that protein levels of rgf1-PH are severely reduced; therefore it is difficult to draw a clear conclusion from this type of assay.

Answer:

To address this issue, we expressed *rgf1*ΔPH-GFP under the control of the actin promoter (pact-*rgf1*ΔPH-GFP). We demonstrated that the expression level of the pact-*rgf1*ΔPH-GFP construct is similar to that of Rgf1-GFP (WT) (Fig S4B). However, pact-*rgf1*ΔPH-GFP does not localize correctly at the cell poles and fails to localize Tea4 or activate Rho1 efficiently (Fig S4C-E). This indicates that the defects observed in the *rgf1*ΔPH mutant are not due to the low amount of protein but rather the absence of the PH domain.

The text in page 14 and line 363 of the revised manuscript has been modified as follows:

"However, in the *rgf1*ΔPH-GFP mutant, the ability to bind and anchor Tea4 to the cortex and to activate Rho1 could be reduced due to a significant drop in the protein level compared with the Rgf1-GFP protein (Fig 3H). To determine if the instability of the *rgf1*ΔPH protein is behind the defects observed in the mutant, we replaced the *rgf1* promoter with that of actin (*act1*), achieving levels of the truncated protein similar to those of the wild-type (Fig S4B). Even so, pact-*rgf1*ΔPH-GFP does not localize correctly at the cell poles and also fails to localize Tea4 or activate Rho1 efficiently (Fig S4C, D and E), indicating that the defects in the *rgf1*ΔPH mutant are not due to the low amount of protein but rather the absence of the PH domain."

We have included a new Fig. S4 B, C, D, E and G, and the corresponding legends (page 41, line 1055).

"(B) Protein extracts from cells producing *rgf1*$^+$-GFP, *rgf1*ΔPH-GFP and pact-*rgf1*ΔPH-GFP were analysed by western blot with an anti-GFP antibody to visualize Rgf1 levels. An anti-tubulin antibody was used as a loading control. (C) Extracts from cells producing Rho1-HA (pREP4X-Rho1-HA) in the WT, *rgf1*Δ, *rgf1*ΔPH and pact-*rgf1*ΔPH-GFP cells were pulled down with GST-C21RBD and blotted against anti-HA antibody (Rho1-GTP). Total Rho1-HA was visualized by western blot (WCE). The relative units indicate the fold-differences in Rho1 levels in the mutants compared with the WT strain, with an assigned value of 1 (bottom) from two independent experiments. (D) Maximum-intensity projection of six Z-slides (0.5 µm step-size) of representative fluorescence images of WT, *rgf1*ΔPH-GFP and pact-*rgf1*ΔPH-GFP cells. (E) The graphic represents the mean ± SD of the relative fluorescence intensity of Tea4-GFP (n > 120) measured at the cellular tips of the WT, *rgf1*ΔPH and pact-*rgf1*ΔPH. Statistical significance was calculated using a two-tailed unpaired Student's t test. (F) Representative images of Rgf1-GFP and Rgf1ΔPTTR-GFP localization. Cells were stained with calcofluor white (20 µg/mL) to spot areas of growth. Scale bar 2 µm. (G) The percentage of cells WT, *tea1*Δ, *rgf1*Δ, *rgf1*ΔPH, pact-*rgf1*ΔPH and *rgf1*ΔPH-ΔPTTR cells forming branches 3 hours after release to growth after 3 days in stationary phase, in the absence and in the presence of MBC (50 µg/ml). The mean ± SD of > 200 cells from three independent experiments is shown. Statistical significance of each strain compared to the wild type was calculated using a two-way ANOVA test. ****P< 0.0001; *P < 0.05; ns = non-significant."

Fig S4B

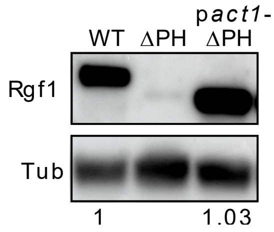

Fig S4C

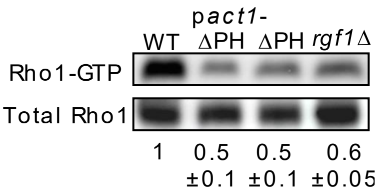

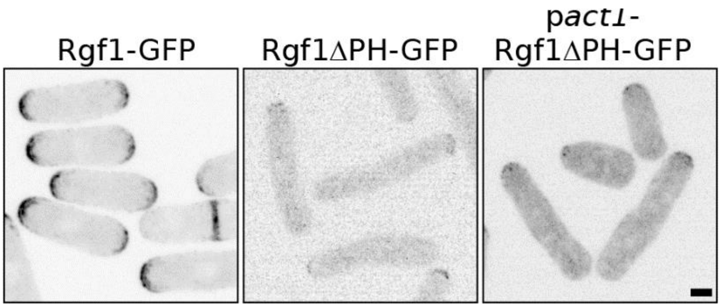

Fig. S4D

Fig S4E

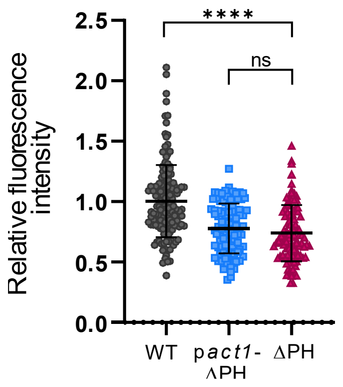

Fig S4G

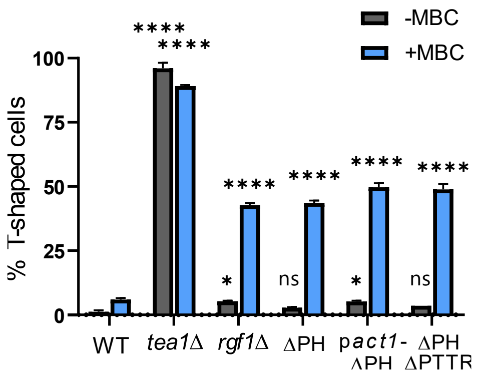

In Figure 4E, the authors state that Rgf1 is required to maintain Tea4 at the poles, and when its activity is impaired, Tea4 levels are reduced, leading to a significant increase in T-shaped cells in the presence of MBC. Tea4-null cells should be included in this analysis, as previously suggested for Figure 2B. Also, it would be interesting to analyze whether artificial targeting of Tea4 to the poles in the absence of rgf1 restores the wild-type phenotype.

Answer:

We have considered both suggestions. We included *tea4*Δ cells in the analysis shown in Fig. 4E and modified Fig. 4E legend accordingly.

Fig 4E

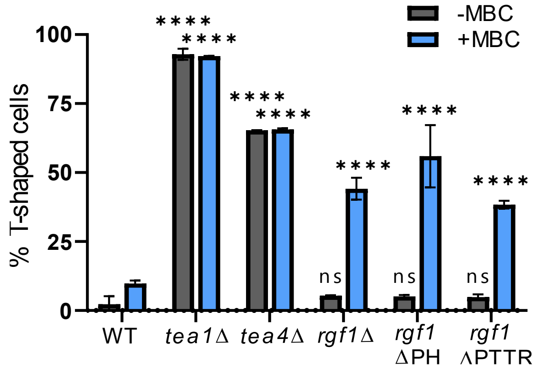

Additionally, we artificially targeted Tea4 to the poles by adding a GBP domain to bind it to the CRIB-GFP protein in a *rgf1*Δ background. Although Tea4 was well positioned at the poles, it did not restore the *rgf1*Δ defects observed in refeeding experiments (Fig 4F).

In the text of the revised manuscript, page 17 and line 420, we have included:

"Furthermore, artificially targeting of Tea4 to the poles by adding a GBP domain to bind it to the CRIB-GFP protein does not restore the *rgf1*Δ defects observed in refeeding experiments (Fig 4F). In sum, these results indicate that Rho1 activation is important for promoting the formation of a stable critical mass of Tea4 in the cell cortex, which is essential to activate pole-restricted growth".

Additionally, we have added Fig. 4F and its corresponding legend (page 16, line 401).

(F) Fluorescence images from log-phase *rgf1*Δ cells expressing CRIB-GFP (green) and Tea4-CBP-mCherry (red). The maximum-intensity projection of six Z-slides (0.5 µm step-size) is shown. Scale bar 2 µm. The graph represents the percentage of cells forming branches after refeeding in the indicated strains. The cells were grown to the stationary phase for 3 days, and then were treated with DMSO (-MBC) or MBC (+MBC; 50 µg/ml) in fresh medium for 3 hours. The mean ± SD of > 200 cells from two independent experiments is shown. Statistical significance was calculated using a two-way ANOVA test. ****P < 0.0001; ***P < 0.001; ns = non-significant.

Fig 4F

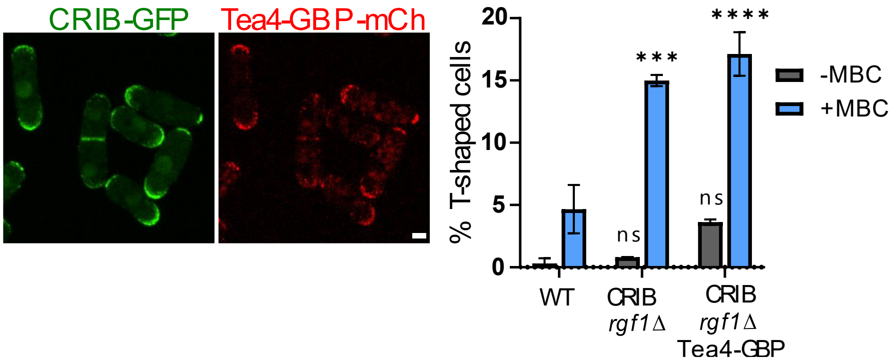

5. Rgf1 is part of actin-dependent machinery that signals growth poles in the absence of the Tea1-Tea4 complex (Figure 5). The authors demonstrate that Rgf1 control of cell polarity acts in parallel to the Tea1-Tea4 complex and suggest that it might involve actin, although the specific molecular mechanisms remain unclear. According to previous work by the authors, in the absence of Rgf1, actin cables are mostly normal and actin patches concentrate at the growing tip. These abnormalities do not exactly match the perturbations on the actin cytoskeleton imposed by stressors or during stationary phase. There might be some overinterpretation of the data.

Answer:

We have analyzed in detail the organization of the actin cytoskeleton in the *rgf1*Δ mutant using LifeAct to observe actin *in vivo* with a more powerful confocal microscope than the fluorescence microscope previously used. We observed that in addition to deficient actin localization at the non-growing pole, a large proportion of *rgf1*Δ cells show actin patches throughout the cell interior, far from the poles where they normally accumulate in wild type cells. This observation indicates a deficiency in actin polarization in the absence of *rgf1*$^{+}$, similar to what is observed after stress. This result, reinforced by the universal role of Rho GTPases as regulators of the actin cytoskeleton, suggests that poor activation of Rho1 affects the organization of the actin cytoskeleton. Although these defects may not be very evident through microscopy, their effects can be detected, especially when the Tea1-Tea4 dependent pathway is eliminated, resulting in a greater number of cells with a T-shaped morphology.

We have included this result in page 19 and line 477:

"Previously, we described that *rgf1*Δ cells show defects in actin reorganization during NETO (28), however other defects in actin patches and cables distribution were not taken account at that time. Now we analyzed the distribution of actin patches along the cell using confocal microscopy and LifeAct to mark actin. Our observations revealed a higher concentration of actin patches in the middle zone of the cell in the absence of *rgf1*, compared to the almost complete absence of

these patches in the wild-type (Fig. S5C), suggesting that *rgf1Δ* cells also exhibit defects in the proper polarization of actin patches. Then, we reasoned…"

Additionally, we have added Fig. S5C and its corresponding legend (page 43, line 1083).

"(C) Images of WT and *rgf1Δ* cells expressing LifeAct-GFP to visualize actin. Maximum-intensity projection of cells (n=22) of the same size from each strain is included to show the distribution of actin patches along the cells."

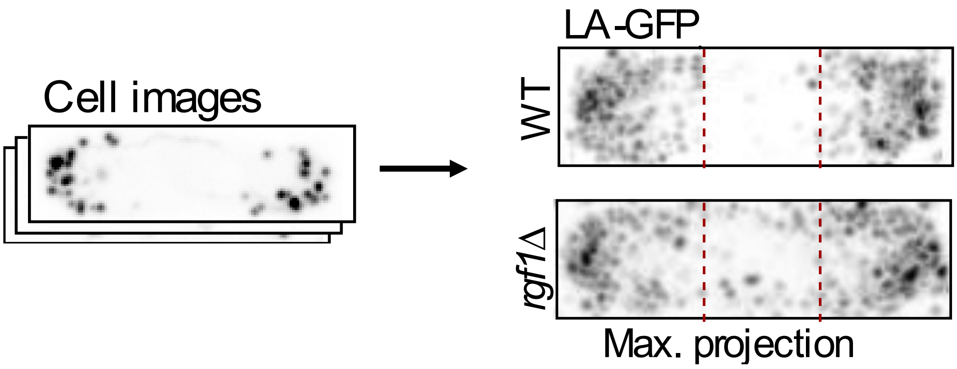

Page 24, line 614, Discussion section:

"The interdependence of actin and Rgf1 localization (Fig 5C and S5D) (30) suggests…"

Page 26, line 660) Discussion section:

"Similarly, when actin becomes disorganized, for example, in the transition from monopolar to bipolar growth (NETO) or in *rgf1Δ* mutant (defective in actin polarization), cells…"

Figure 5A. The description of these results is not clear. The authors indicate that stress treatments also induced branching in tea1-null mutant for 3 days in liquid medium, but it is not entirely clear if this is what is shown in panel A. In this panel, stationary cells are displayed, and it is noted that they have not been treated with stressors.

Answer:

We consider that the starvation that occurs during stationary phase is a stress situation for the cells. It triggers various adaptive responses and metabolic shifts aimed at survival and maintenance. We apologize because a sentence in the manuscript very close to the explanation of Fig 5A was misplaced. In the revised version we have corrected the mistake so that the explanation of Fig 5A is placed appropriately, ensuring clarity and coherence in the presentation.

In the text of the revised manuscript page 17 and line 434 we have included:

"To address this issue, we confirmed the disorganization of the actin cytoskeleton in wild-type cells grown for 3 days in liquid medium (stationary phase, Fig 5A). In addition, exposure to KCl, sorbitol or heat, which promoted actin depolarization in wild type cells also induced branching in the *tea1Δ* mutant (Fig S5A and B) (11,51)"

Figure 5G-I. LatA treatment disassembles both actin patches and cables, which contribute to endocytosis and transport, respectively. Specifying which actin structures are relevant in this

control is important since both contribute to polarized growth, and this information is lacking in the manuscript.

Answer:

We followed the referee's suggestions. For the analysis of actin patches impact, wild-type cells were treated with CK666 for 2 hours, washed and exposed to MBC for 4 hours (to remove MTs). For the analysis of actin cables contribution, *for3Δ* cells were treated with MBC. The results shown in Fig. S5K, indicate that both cables and patches similarly contribute to polarized growth in the absence of microtubules.

We have included these results in page 22 and line 553

"Interestingly, cables and patches similarly contribute to polarized growth in the absence of microtubules (Fig. S5K)"

and on Figure S5K and the figure legend (page 44, line 1106)

Fig S5: "(K) Quantitation of the T-shaped cells in wild-type cells treated with DMSO (WT DMSO), MBC 50 µg/mL (WT MBC) for 4 hours, 100 µM of CK666 for 2 hours to eliminate actin patches and then washed and allowed to grow with DMSO (WT CK) or 50 µg/mL of MBC for 4 hours (WT CK-MBC). *for3Δ* cells, which lack actin cables, were treated with DMSO (*for3Δ* DMSO) or MBC 50 µg/mL (*for3Δ* MBC) for 4 hours. Wild type cells treated with 50 µM of LatA for 2 hours, were washed, and then treated with 50 µg/mL of MBC for 4 hours (WT LatA-MBC). The graph represents the mean ± SD of > 200 cells from three independent experiments. Statistical significance was calculated using a two-way ANOVA test. ****P < 0.0001; **P < 0.01; ns = non-significant. Scale bar 2 µm."

Fig S5K

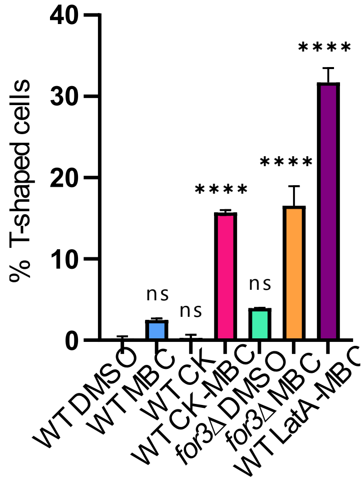

To complete this analysis, I will also include additional regulators of Cdc42, such as Scd1 and Scd2 (GEFs for Cdc42).

We have attempted to create double mutants *scd1Δtea1Δ* and *scd2Δtea1Δ*, but encountered significant challenges due to the decreased mating efficiency exhibited by both *scd1* and *scd2*

mutants. Additionally, these mutants display a rounded cell morphology, which complicates the detection of branching in the absence of *tea1*.

Rev. 2:

This manuscript examines cell polarity in the fission yeast *S. pombe*. Cell tip growth has been known to depend on the Tea1-Tea4 complex that is delivered to cell tips by dynamic microtubules. In this paper, the authors propose that Rho1-GTPase activity, under control of the GEF Rgf1 and the actin cytoskeleton, serve as a secondary signal for cell tip growth. A combination of microscopy and biochemistry demonstrate that Rgf1 interacts with Tea4 and is required for stable localization of Tea4 at cell tips. Combined loss of Rgf1 and Mod5, which also stabilizes Tea1-Tea4 at tips, leads to dramatic T-shaped cells. This activity of Rgf1 might depend on its membrane-binding domain, although these data are not strong (see below). The authors also define regulation of the Rgf1-Rho1 pathway during stress conditions, which have been known to impact the Tea1-Tea4 pathway and its mutant phenotypes. Overall, the paper represents a significant contribution for the field because it identifies an actin-based cell polarity pathway that functions in parallel with the previously defined microtubule-based cell polarity pathway. Most of the data are very strong, with several exceptions that are described below and can be fixed. The work will draw interest for researchers studying cell polarity and stress signaling in many cell types, and the themes of this work will likely apply broadly.

Specific comments:

1. The authors show that Tea4 localization at cell tips is unstable in rgf1Δ mutants, similar to mod5Δ and tea3Δ mutants. This result begs the question if Mod5 and Tea3 are properly localized in rgf1Δ mutants. It seems possible that Rgf1 acts on Tea4 through these other anchoring proteins, although the genetic data also support the possibility of parallel action by multiple anchors. Therefore, testing Mod5 and Tea3 localization in rgf1Δ cells is interesting either way.

Answer.

Thank you for your suggestion. We have analyzed Mod5 and Tea3 localization by measuring mEGFP-Mod5 and Tea3-GFP fluorescence intensity in wild type and *rgf1Δ* cells at both the growing and non-growing poles (see below, Fig. 4, not included). Similar to the wild type, differences between the poles were observed in both strains, with Mod5 and Tea3 being more concentrated at the non-growing end. However, when considering the growing end alone or the non-growing end alone, we did not observe differences in the localization between the wild type and the *rgf1Δ* mutant. Therefore, Tea4 anchoring in the absence of Rgf1 does not depend on the ability of Rgf1 to anchor Mod5 and Tea3.

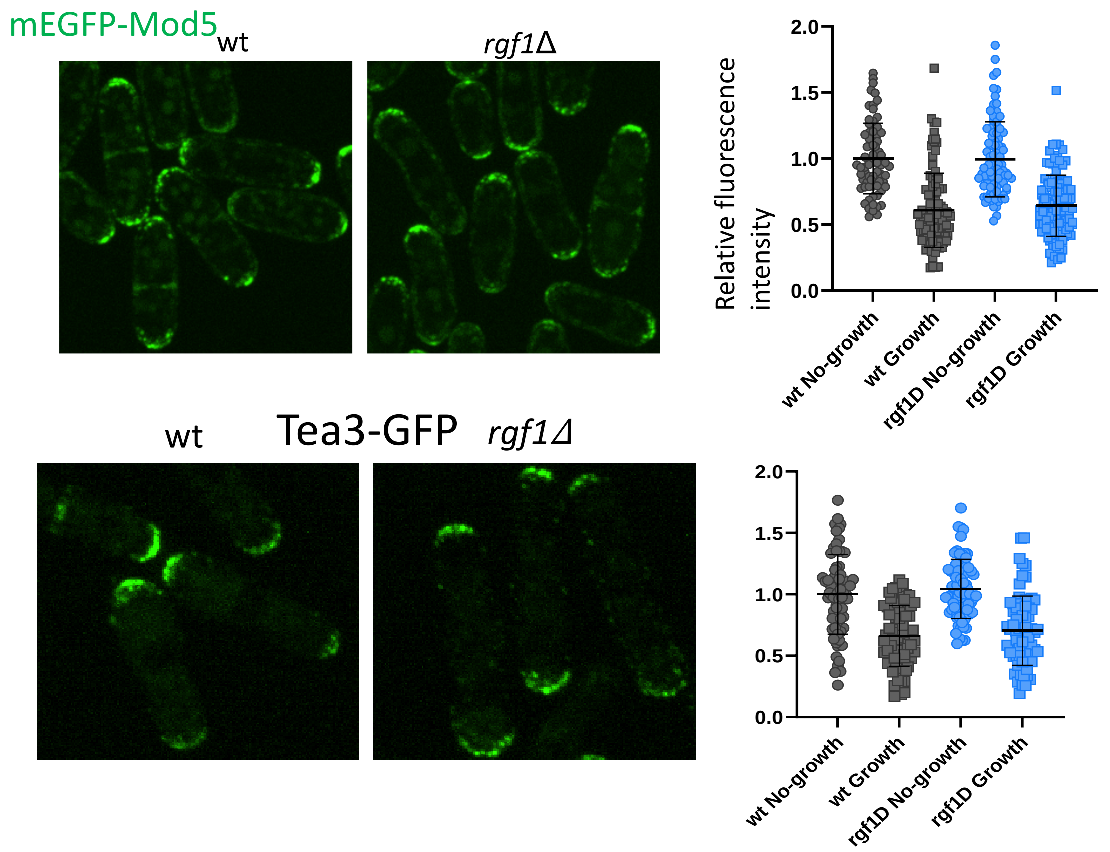

Fig. 4. GFP fluorescence in cells expressing *mEGFP-mod5 rgf1*$^{+}$ and *mEGFP-mod5 rgf1*Δ (upper) or *tea3-GFP rgf1*$^{+}$ and *tea3-GFP rgf1*Δ (lower). The maximum-intensity projection of six Z-slides (0.5 µm step-size) of GFP fluorescence is shown. The graphs represent the mean ± SD of the relative fluorescence intensity of mEGFP-Mod5 or Tea3-GFP measured at the growing end and non-growing end of the WT and *rgf1*Δ (n > 70) cells. Calcofluor staining, which marks sites of cell growth, was used to differentiate growth from non-growth poles.

2. The authors claim that Rgf1 dots and Tea4 dots colocalize, but the images in Figure 3A are not strong enough to support this conclusion. I would strongly suggest that the authors use their innovative "head-on" imaging (as in Figure 1F) to test if Rgf1-tdT and Tea4-GFP dots colocalize at cell tips.

As suggested, Super-Resolution Radial Fluctuations (SRRF) microscopy for head-on imaging of cell tips for Tea4 and Rgf1 co-localization is now included in Figure 3A, with a conventional SRRF side image in Figure S3A. In the new Figure 3A, a partial colocalization between Tea4 and Rgf1 can be observed more clearly, as a large number of points for both proteins coincide, although not all of them. Moreover, we cannot rule out that the points that do not show complete overlap are due to the lag in image capture for each channel.

We have included these results in page 11 and line 276.

“First, we examined the *in vivo* localization of endogenous Tea4-GFP together with Rgf1-tdTomato at the cell poles by SRRF microscopy for “head-on” imaging of cell tips. In interphase cells….”

Fig. 3A and Fig. S3A and in the corresponding figures legends.

Page 12, line 295. Fig. 3: "(A) Colocalization of Rgf1 and Tea4. Super-Resolution Radial Fluctuations (SRRF) images in "head-on" cell tips of the wild-type producing Tea4-GFP endogenously (green) and Rgf1-tdTomato from a plasmid under the control of its own promoter (red)."

Page 41, line 1044. Fig. S3: "(A) Colocalization of Rgf1 and Tea4. Super-Resolution Radial Fluctuations (SRRF) images of wild-type cells producing Tea4-GFP endogenously (green) and Rgf1-tdTomato from a plasmid under the control of its own promoter (red)."

Fig 3A

Tea4-GFP Rgf1-toma SRRF

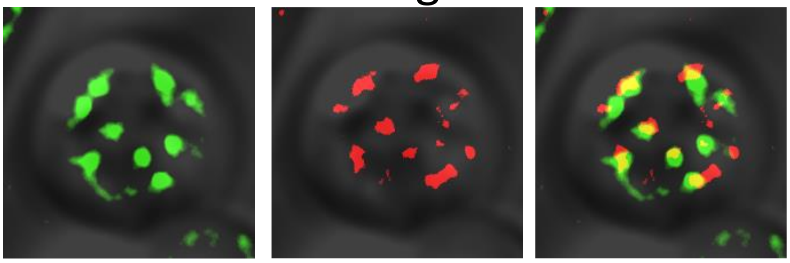

Fig S3A

Tea4-GFP Rgf1-toma SRRF

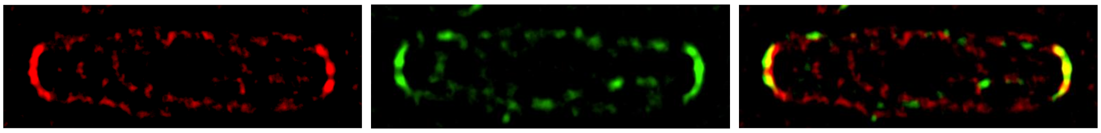

3. Figure 3C: This western blot shows results for co-immunoprecipitation assays for Rgf1 interactions with Tea1 and Tea4. I am confused why/how Tea1-GFP and Tea4-GFP are shown at the same molecular weight in the gels. They have different molecular weights (Tea1 is 127 kDA, and Tea4 is 90 kDa), so they should migrate as differently sized bands by SDS-PAGE.

Answer:

This is intriguing. According to Pombase, Tea1's molecular weight (Mw) is about 127.44 kDa, and Tea4's Mw is ~90.79 kDa (https://www.pombase.org/gene). We have tested the strains by PCR to ensure that we have the GFP over the right gene. In our co-immunoprecipitation assays, we used gradient gels, but we specifically ran a 6% acrylamide gel for an extended period to detect differences between Tea1-GFP and Tea4-GFP. In our hands, both proteins tagged with GFP run alike at ~180 kDa (Fig. 5), but Tea4-GFP moves slightly faster than Tea1-GFP. Additionally, Tea1 appears more abundant than Tea4, which is consistent with the protein levels detected by Marguerat et al. (2012) in vegetative growing cells. They detected 1910 molecules per cell of Tea1 compared to only 332 molecules of Tea4.

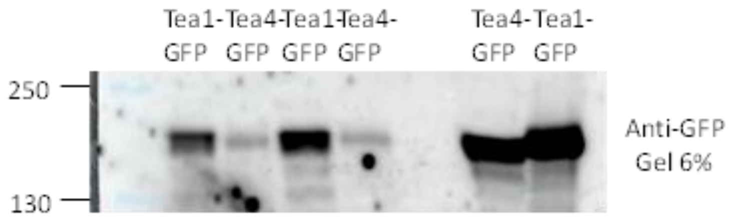

Fig. 5. Protein extracts from wild type cells expressing Tea1-GFP or Tea4-GFP were visualized by western blot with antibodies against GFP.

In the literature, it has been observed that Tea4-Flag runs between 100 and 150 kDa (~130 kDa) (Koyano et al., 2016; DOI:10.1111/gtc.12309). Tea4 carries a Src homology 3 (SH3) domain at residues 121–178 and a cluster of acidic amino acid residues at positions 81–99. The rest of the protein seems to contain mostly unstructured or disordered regions, which could account for its unusual migration properties in acrylamide gels. Additionally, this result might be due to post-translational modifications that affect the migration of these proteins in SDS-PAGE, leading to similar migration patterns despite their differing predicted molecular weights. Further investigations are needed to fully understand the cause of this anomalous migration.

4. Figure 3D: For the yeast two-hybrid assays, the authors need to include the negative control of Tea4 and Tea1 alone (it would be -/Tea4 and -/Tea1 by their nomenclature). This would control for autoactivation by these constructs.

In the revised version it has been included a new Fig. 3D, with negative controls for the interaction between Tea4/Rgf1 (Tea4/empty plasmid) as well as for the interaction Tea1/Rgf1 (Tea1/empty plasmid). In both cases, no growth was observed.

Page 12 and line 307, the legend of Fig. 3D has been modified accordingly:

"The interaction was assessed on YNB plates without histidine (YNB -H), including Rgf1/pGAD (Rgf1/-), Tea1/pGBK (-/Tea1) and Tea4/pGBK (-/Tea4) as controls."

Figura 3D

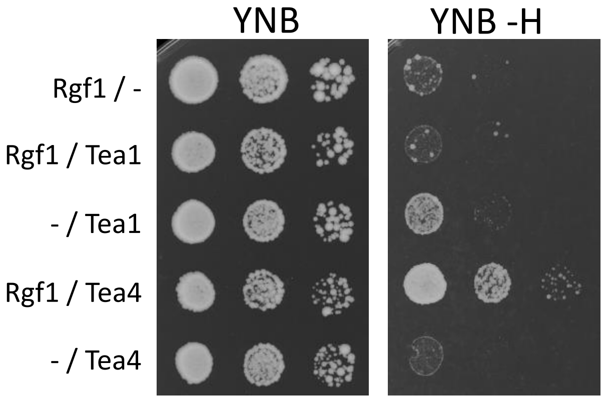

5. The authors make a rgf1ΔPH mutant that lacks the lipid-binding domain and observe defects in this mutant. However, the mutant is expressed at massively reduced levels compared to wildtype (as shown in Figure 3H and mentioned in the text). The reduced expression makes it impossible to interpret any role of membrane binding, although the authors conclude that

membrane-binding is important for cell tip localization and function. I would argue that protein expression is important for cell tip localization, since the low concentration will reduce the levels at cell tips. I would also argue that phenotypic defects for this mutant are due to its extremely low levels. It seems very important for the authors to test this construct under increased expression levels, so that its levels are similar to wildtype. They can perform this experiment with an exogenous promoter. Otherwise, I disagree that the mutant has compromised localization and function. Importantly, this problem extends to other parts of the paper as well because the authors use the same under-expressed mutant to test Rho1 activation, Tea4 binding, cell branching, and more. In my opinion, all of these experiments need to be repeated under increased expression of Rgf1ΔPH.

Answer:

Thank you for the suggestion, we expressed *rgf1*ΔPH-GFP under the control of the actin promoter (pact-*rgf1*ΔPH-GFP). We demonstrated that the expression level of the pact-*rgf1*ΔPH-GFP construct is similar to that of Rgf1-GFP (WT) (Fig S4B). However, pact-*rgf1*ΔPH-GFP does not localize correctly at the cell poles and fails to localize Tea4 or activate Rho1 efficiently (Fig S4C-E). This indicates that the defects observed in the *rgf1*ΔPH mutant are not due to the low amount of protein but rather the absence of the PH domain.

The text (page 14, line 363) of the revised manuscript has been modified as follows:

"However, in the *rgf1*ΔPH-GFP mutant, the ability to bind and anchor Tea4 to the cortex and to activate Rho1 could be reduced due to a significant drop in the protein level compared with the Rgf1-GFP protein (Fig 3H). To determine if the instability of the *rgf1*ΔPH protein is behind the defects observed in the mutant, we replaced the *rgf1* promoter with that of actin (*act1*), achieving levels of the truncated protein similar to those of the wild-type (Fig S4B). Even so, pact-*rgf1*ΔPH-GFP does not localize correctly at the cell poles and also fails to localize Tea4 or activate Rho1 efficiently (Fig S4C, D and E), indicating that the defects in the *rgf1*ΔPH mutant are not due to the low amount of protein but rather the absence of the PH domain."

The text (page 42, line 1055) we have included a new Fig. S4 B, C, D, E and G and the corresponding legends.

"(B) Protein extracts from cells producing *rgf1*$^{+}$-GFP, *rgf1*ΔPH-GFP and pact-*rgf1*ΔPH-GFP were analysed by western blot with an anti-GFP antibody to visualize Rgf1 levels. An anti-tubulin antibody was used as a loading control. (C) Extracts from cells producing Rho1-HA (pREP4X-Rho1-HA) in the WT, *rgf1*Δ, *rgf1*ΔPH and pact-*rgf1*ΔPH-GFP cells were pulled down with GST-C21RBD and blotted against anti-HA antibody (Rho1-GTP). Total Rho1-HA was visualized by western blot (WCE). The relative units indicate the fold-differences in Rho1 levels in the mutants compared with the WT strain, with an assigned value of 1 (bottom) from two independent experiments. (D) Maximum-intensity projection of six Z-slides (0.5 µm step-size) of representative fluorescence images of WT, *rgf1*ΔPH-GFP and pact-*rgf1*ΔPH-GFP cells. (E) The graphic represents the mean ± SD of the relative fluorescence intensity of Tea4-GFP (n > 120) measured at the cellular tips of the WT, *rgf1*ΔPH and pact-*rgf1*ΔPH. Statistical significance was calculated using a two-tailed unpaired Student's t test. (F) Representative images of Rgf1-GFP and Rgf1ΔPTTR-GFP localization. Cells were stained with calcofluor white (20 µg/mL) to spot areas of growth. Scale bar 2 µm. (G) The percentage of cells WT, *tea1*Δ, *rgf1*Δ, *rgf1*ΔPH, pact-*rgf1*ΔPH and *rgf1*ΔPH-ΔPTTR cells forming branches 3 hours after release to growth after 3 days in stationary phase, in the absence and in the presence of MBC (50 µg/ml). The mean ± SD of > 200 cells from three independent experiments is shown. Statistical significance of each strain compared to the wild type was calculated using a two-way ANOVA test. ****P< 0.0001; *P < 0.05; ns = non-significant.

Fig S4B

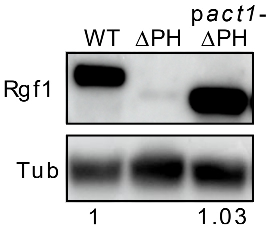

Fig S4C

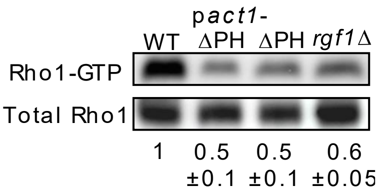

Fig.S4D

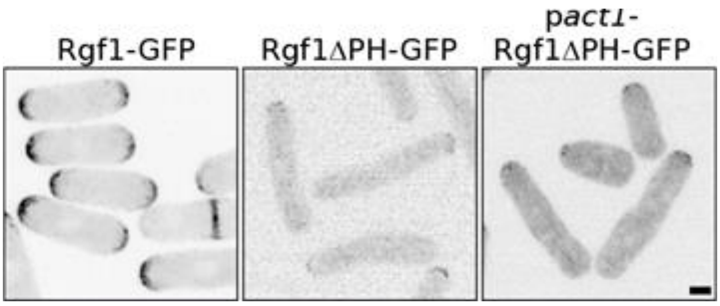

Fig S4E

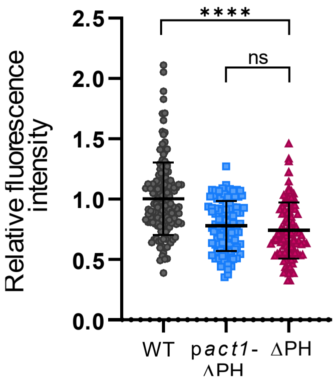

Fig S4G

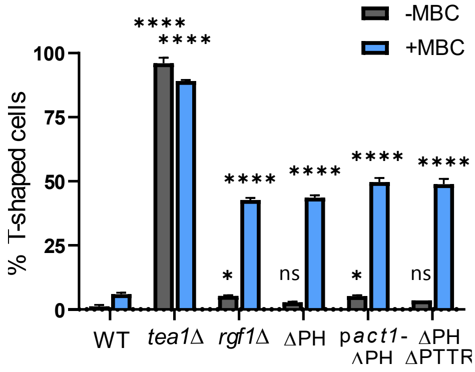

6. Given the previous point, it would be helpful for the authors to validate that Rgf1-ΔPTTR mutant is expressed as similar levels as wild type Rgf1.

Answer:

This is shown in a previous paper from the lab (Garcia *et al*. 2009, DOI: 10.1091/mbc.e08-07-0673). In this paper, we tagged the full-length wild type and the mutant *rgf1-PTTRΔ* gene with GFP at the carboxy-end. These proteins were expressed at comparable levels as it is shown in Fig 4D (WCE).

7. An alternative interpretation of the synthetic defects in rgf1Δ tea4Δ cells is that rgf1Δ induces cell stress. Most of the phenotypes examined for tea1Δ and tea4Δ cells are exacerbated by cell stress or by rgf1Δ. The authors might consider this possibility in their Discussion section. It would be a less exciting model, but nonetheless seems plausible from the available data.

Answer:

We have addressed the reviewer's concern by examining the role of the MAP kinase Sty1 pathway in this context. Our findings suggest that the absence of Rgf1 does not act as a stressor that activates the Sty1 pathway, nor does this pathway play a significant role in the polarity defects observed in the *rgf1Δ tea1Δ* double mutant. We hope this addresses the reviewer's concern and provides a clear understanding of the relationship between Rgf1, the Sty1 pathway, and the observed phenotypes in our mutants. Here is the revised and expanded explanation for our findings:

In Page 21, line 522 it has been included the following paragraph

"Because stresses that induce actin disorganization and Rgf1 delocalization at the poles increase the percentage of T-shaped cells in the tea1Δ mutant (Fig 5D 5E, S5A and S5AS5B), we asked whether the absence of Rgf1 by itself acts as a stressor, thereby increasing the number of branched cells in the double mutant. In this regard, it has been demonstrated that treatment with LatA causes a delocalization of proteins necessary for polarized growth, such as the active form of Cdc42. The dispersal of Cdc42 from growth sites depends on the activation of MAP kinase Sty1 while it is independent of actin delocalization per se (52,54). Our group described years ago that while Rgf1 stimulates Pmk1 MAPK pathway activity in response to certain stresses, it was not involved in the activation of Sty1, neither under stress nor in basal conditions (38). Accordingly, we observed no increase in the nuclear localization of Sty1, nor in the phosphorylation of the transcription factor Atf1 in the *rgf1Δ* mutant (Fig S5G and H), as would be expected if Sty1 were active (55). Unlike delocalization of the active form of Cdc42 (marked by CRIB-GFP) (52,54), Rgf1 delocalization after stress does not depend on Sty1 (Fig S5I). Moreover, the number of cells forming T-shapes in the absence of both Rgf1 and Tea1 does not decrease upon the elimination of Sty1 (Fig S5J). Taken together, these results indicate that the absence of Rgf1 does not activate the Sty1 pathway (stress activated pathway, SAP), nor is the SAP important for the loss of polarity that occurs in the rgf1Δ tea1Δ double mutant.

An alternative explanation for the observed increase in branched cells when both Rgf1 and Tea1 were absent is that Tea1 and Rgf1 participate in parallel pathways to define the sites of cell growth, each one necessary when the other is not present. Thus, we wondered"

We have included Fig S5G, H, I and J, and the corresponding figure legends (page 43, line 1095):

"(G) GFP fluorescence in WT and *rgf1Δ* log-phase cells expressing *sty1-GFP.* Cells either were untreated (upper panels) or treated with 1 mM H_2O_2 for 15 min (lower panels). The maximum-intensity projection of six Z-slides (0.5 µm step-size) of Sty1-GFP fluorescence is shown. (H) Extracts from wild type (WT-unt) and *rgf1Δ* cells (*rgf1Δ*-unt) were compared with extracts from WT cells treated with 1 mM H_2O_2 for 15 min. Proteins were visualized by western blot with antibodies against Atf1. (I) GFP fluorescence in WT and *sty1Δ* log-phase cells expressing *Rgf1-GFP*. Cells were either untreated (upper panels) or treated with 0.6 M KCl for 1 hour (lower panels). The maximum-intensity projection of six Z-slides (0.5 µm step-size) of Rgf1-GFP fluorescence is shown. (J) The percentage of T-shaped cells (n >200) in the indicated strains grown to log phase in YES liquid medium at 28°C. Statistical significance was calculated using a two-tailed unpaired Student's t test. ns = non-significant. The experiment was repeated 4 times."

Fig. S5G

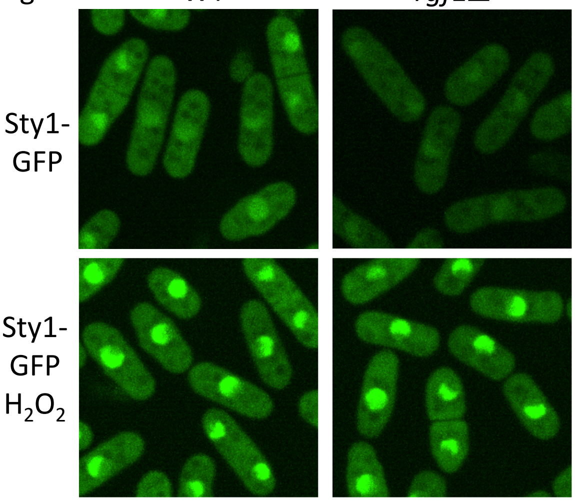

Fig. S5H

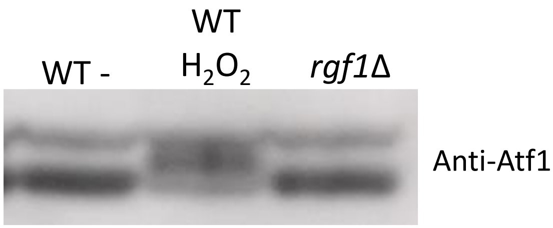

Fig. S5I

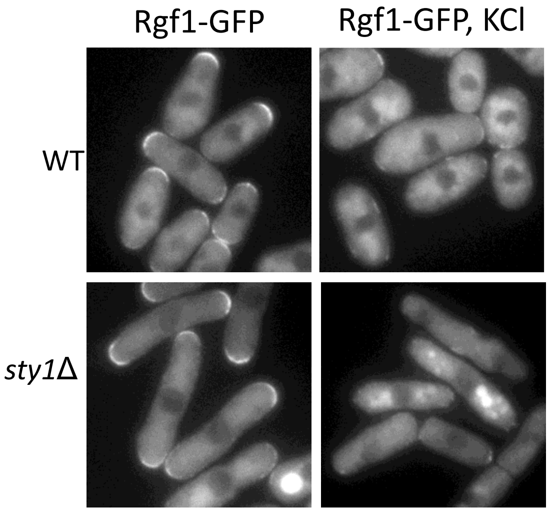

Fig S5J

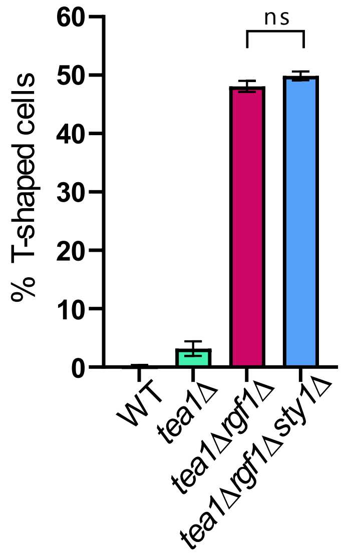

8. The authors propose feedback between Rgf1 and the actin cytoskeleton. This is an interesting idea, but I do not see evidence that Rgf1 regulates the actin cytoskeleton. Are there actin defects beyond monopolar growth in rgf1Δ mutants? Or alternatively, does the actin-Rgf1 relationship act in one direction with actin upstream of Rgf1?.

Answer:

We have analyzed in more detail the organization of the actin cytoskeleton in the *rgf1Δ* mutant using LifeAct to observe actin in vivo with a more powerful confocal microscope than the fluorescence microscope previously used. We observed that in addition to deficient actin localization at the non-growing pole, a large proportion of *rgf1Δ* cells show actin patches throughout the cell interior, far from the poles where they normally accumulate in wild-type cells. This observation indicates a deficiency in actin polarization in the absence of $rgf1^{+}$, similar to what is observed after stress in the wild type strain. This result, reinforced by the universal role of Rho GTPases as regulators of the actin cytoskeleton, suggests that poor activation of Rho1 affects the organization of the actin cytoskeleton. Although these defects may not be very

evident through microscopy, their effects can be detected, especially when the Tea1-Tea4 dependent pathway is eliminated, resulting in a greater number of cells with a T-shaped morphology.

It is very difficult to separate the functions of actin and Rgf1 as they are interdependent. On one hand, Rgf1 requires the actin cytoskeleton to reach the growth regions of the plasma membrane (Fig S5D). We have seen that the correct localization of Rgf1 is essential for Rho1 activation (Fig 3F and 4B), so if Rgf1 is not properly localized Rho1 becomes nonfunctional. On the other hand, actin also seems to depend on Rgf1 and Rho1 for its own organization, as in the absence of Rgf1 activity, actin is less polarized, which could be important for its function. We cannot completely rule out the hypothesis that actin functions upstream of Rgf1-Rho1, but it seems more plausible to us that both actin and Rho1 are responsible for defining the growth sites in the absence of Tea markers.

We have included this result in page 19 and line 477:

"Previously, we described that rgf1Δ cells show defects in actin reorganization during NETO (30), however other defects in actin patches and cables distribution were not taken account at that time. Now we analyzed the distribution of actin patches along the cell using confocal microscopy and LifeAct to mark actin. Our observations revealed a higher concentration of actin patches in the middle zone of the cell in the absence of rgf1, compared to the almost complete absence of these patches in the wild-type (Fig. S5C), suggesting that rgf1Δ cells also exhibit defects in the proper polarization of actin patches. Then, we reasoned…"

Additionally, we have added Fig. S5C and its corresponding legend (page 43, line 1083).

"(C) Images of WT and *rgf1Δ* cells expressing LifeAct-GFP to visualize actin. Maximum-intensity projection of cells (n=22) of the same size from each strain is included to show the distribution of actin patches along the cells."

Fig S5C

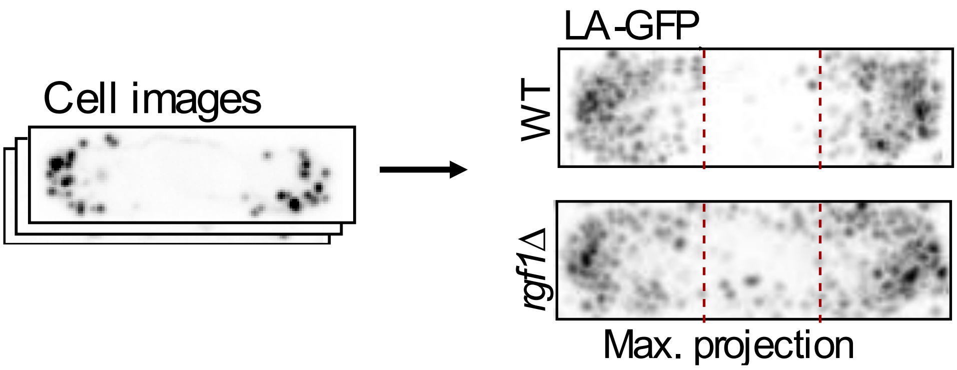

Page 24, line 614, Discussion section:

"The interdependence of actin and Rgf1 localization (Fig 5C and S5D) (28) suggests…"

Page 26, line 660, Discussion section:

"Similarly, when actin becomes disorganized, for example, in the transition from monopolar to bipolar growth (NETO) or in *rgf1Δ* mutant (defective in actin polarization), cells…"

9. At the bottom of page 22, the authors state that Tea4 and Rgf1-Rho1 are essential for each other's cell tip localization. However, I don't recall data for Rgf1-GFP localization in tea4Δ cells. Can the authors clarify if there is a defect?

In the revised version, it has been included in Fig. S5E, time-lapse images of Rgf1-GFP localization in *tea4Δ* cells during recovery from KCl-induced stress, suggesting that Tea4 is required for Rgf1 re-localization to the poles after osmotic stress.

Page 43, line 1090, legend of Fig S5E has been included the following sentence:

"The localization of Rgf1-GFP in *tea4Δ* cells under the same conditions is shown in the lower panel".

Fig S5E

Rgf1-GFP *tea4Δ*

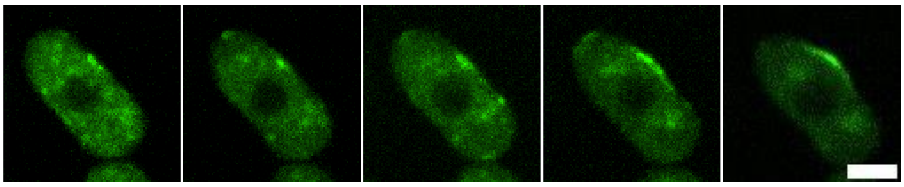

10. Minor: In line 164, the authors state "Tea1 also localized..." but I think they mean "Tea4 also localized..."

Answer:

Ok ...we meant to say Tea4 also localized. However, in the revised version (suggested by one of the reviewers) we have included photographs showing the localization of Tea1-GFP in the wild type and the *rgf1Δ* mutant (Fig S1D). Additionally, we quantified the fluorescence intensity of Tea1-GFP at the poles of both strains comparing the fluorescence intensity of the growing and non-growing poles.

In the text (page 7 and line 173), we have included ...

"Given that in *rgf1Δ* cells, Tea1 also localizes mainly to the non-growing end (Fig S1D), our results suggest that the Tea1 mislocalization could be a consequence of Tea4 mislocalization."

11. Minor: on line 267, there is a typo in Schizosaccharomyces.

This has been fixed.

12. Minor: Figure 5A should be relabeled to spell out "stationary phase" because "S-phase" has a different meaning in the cell cycle.

This has been fixed.

Academic Editor's comments:

This is an interesting manuscript by Sanchez and colleagues.

The first part of the manuscript supports a role for Rgf1 in the Tea1/4 pathway, by acting as an anchor of Tea4 at cell poles. This part of the manuscript is well supported, although it is unclear at present whether Rgf1 acts as physical anchor or acts indirectly (in unknown manner) through activation of Rho1. There are a few issues that would need to be addressed, listed below, but this part is not the main point of the paper.

A) The authors show that Tea4-GFP localization at cell poles is vastly reduced in rgf1Δ mod5Δ double mutants. It would be interesting to test the localization of Tea1-GFP in the rgf1Δ mod5Δ double mutants to check whether Rgf1 functions upstream of Tea1 or in parallel to it to localize Tea4.

Answer:

We analyzed the localization of Tea1 in *rgf1Δ mod5Δ* double mutants and quantified the fluorescence enrichment at the cell tips for these experiments and the results are now included in the text of the revised version. The Tea1 signal at the poles exhibited a pattern similar to that of Tea4 in both single and double mutants.

The text page 9 and line 222 of the revised manuscript has been modified as follows. …

"we analyzed the localization of Tea4 and Tea1 in the double mutant *rgf1Δmod**5**Δ* compared with the single mutant."

Page 9 and line 227. "The Tea1 signal at the poles exhibited a pattern similar to that of Tea4 in both single and double mutants (Fig S2A), suggesting that Mod5 and Rgf1 share a function in Tea1-Tea4 anchoring**."**

We have included a new Fig. S2A and the corresponding Fig. S2A legend.

Fig S2A

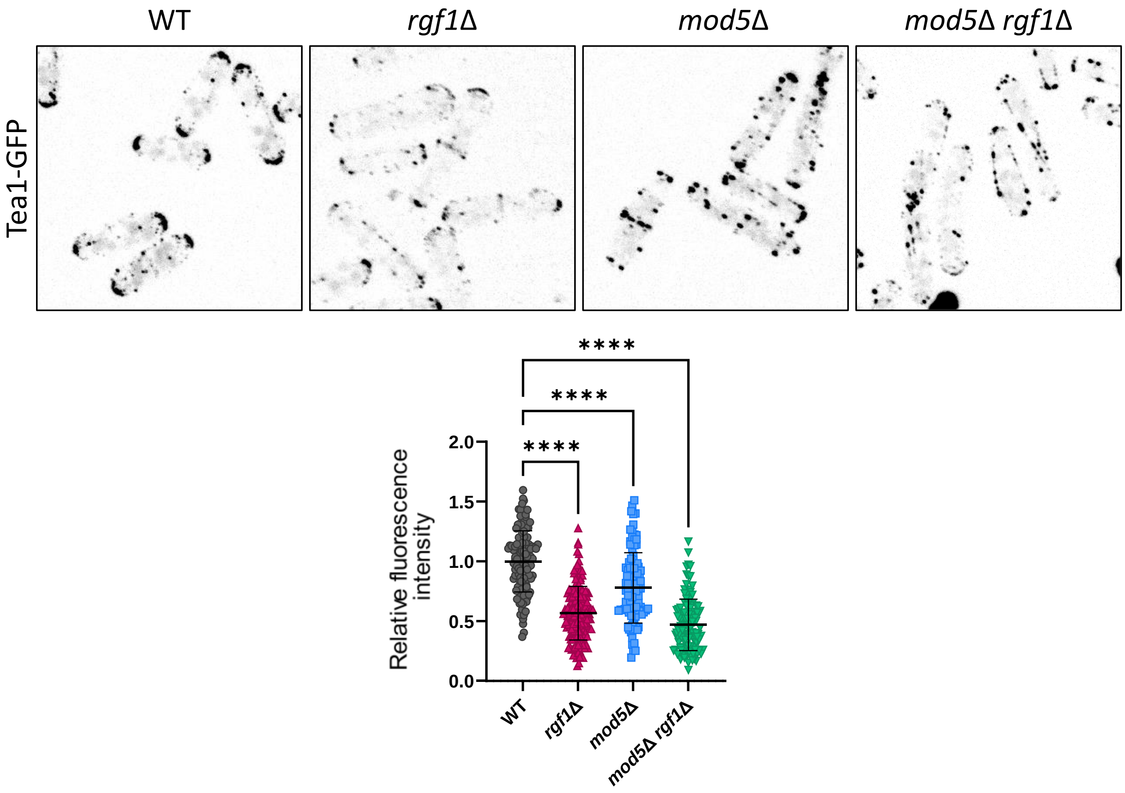

Page 41 and line 1035. Figure S2. "(A) Representative images of the indicated strains producing Tea1-GFP. The maximum-intensity projection of six Z-slides (0.5 µm step-size) is shown. Scale bar: 2 µm. The graph represents the mean ± SD of the relative fluorescence intensity of Tea1-GFP measured at the cell tips in the wild type, *rgf1*Δ, *mod5*Δ and *rgf1*Δ *mod5*Δ cells (n >100). WT levels were used for normalization."

B) The section on the role of Rgf1 activity and localization is a bit overstated. Because the ΔPH mutant is unstable, it is impossible to make strong claims about the role of Rgf1 localization. It could be that the phenotype of this mutant is due to loss of protein stability. Similarly, at the bottom of page 11, it is not possible to claim that "GEF activity is more critical than GEF localization". The data show that GEF activity is critical, but no strong conclusion can be made on the importance of localization.

Answer:

To address the first question, we expressed *rgf1*ΔPH-GFP under the control of the actin promoter (pact-*rgf1*ΔPH-GFP). We demonstrated that the expression level of the pact-*rgf1*ΔPH-GFP construct is similar to that of Rgf1-GFP (WT) (Fig S4B). However, pact-*rgf1*ΔPH-GFP does not localize correctly at the cell poles and fails to localize Tea4 or activate Rho1 efficiently (Fig S4C-E). This indicates that the defects observed in the *rgf1*ΔPH mutant are not due to the low amount of protein but rather the absence of the PH domain.

The text (page 14, line 363) of the revised manuscript has been modified as follows:

"However, in the *rgf1*ΔPH-GFP mutant, the ability to bind and anchor Tea4 to the cortex and to activate Rho1 could be reduced due to a significant drop in the protein level compared with the Rgf1-GFP protein (Fig 3H). To determine if the instability of the *rgf1*ΔPH protein is behind the defects observed in the mutant, we replaced the *rgf1* promoter with that of actin (*act1*), achieving levels of the truncated protein similar to those of the wild-type (Fig S4B). Even so, pact-*rgf1*ΔPH-GFP does not localize correctly at the cell poles and also fails to localize Tea4 or activate Rho1 efficiently (Fig S4C, D and E), indicating that the defects in the *rgf1*ΔPH mutant are not due to the low amount of protein but rather the absence of the PH domain."

We have included new Fig. S4 B, C, D, E and G and the corresponding legend (see response to reviewer 1 and 2).

The second issue is in regard of the statement that "GEF activity is more critical than GEF localization". To address this issue, we have revised the paper to emphasize that both GEF activity and localization are required for maintaining Tea4 at the cell tips and preserving the growth pattern after refeeding.

Page 17 and line 416 of the revised version is as follows:

"Taken together, these results indicate that the localization of Rgf1 to the PM and its ability to activate Rho1 are closely linked; both functions are required to maintain Tea4 at the cell tips and to preserve the growth pattern after refeeding. The elimination of the PH domain and mutation of the catalytic site simultaneously has no additive effect, demonstrating this link (Fig 4A, S4A and S4G). Furthermore, artificially targeting of Tea4 to the poles by adding a GBP domain to bind it to the CRIB-GFP protein does not restore the *rgf1*Δ defects observed in refeeding

experiments (Fig 4F). In sum, these results indicate that Rho1 activation is important for promoting the formation of a stable critical mass of Tea4 in the cell cortex, which is essential to activate pole-restricted growth."

C) It is not clear to me how Rgf1 may function to localize Tea4 at cell poles yet be delocalized to lateral patches upon stresses (Figure 5E), while Tea1/4 isn't. This makes it a bit unlikely that Rgf1 serves to mark the pole. This could be discussed.

First, we have not meant to say that the localization of Tea4 depends entirely on Rgf1. In fact, we have shown that Mod5 is redundant with Rgf1 regarding Tea1/Tea4 localization at the poles. In particular, the localization of Mod5 is not disturbed upon stresses such as stationary phase (Fig. 5D). Furthermore, although Rgf1 delocalizes in the presence of stress, the localization of Tea1 and Tea4 at the poles is less dynamic. This is shown below: Tea4 is maintained at the cell tips after treatment with MBC for 2.5 hours, which clearly depolymerizes microtubules. In addition, MTs may continue depositing polarity markers at the cell poles under these stresses.

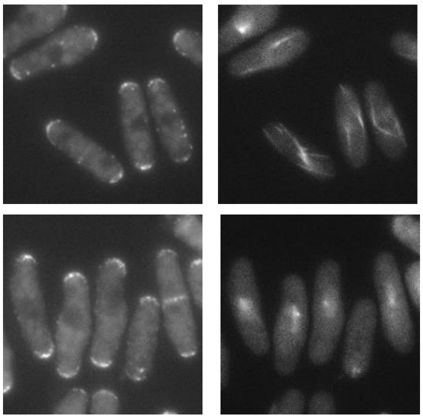

In the second part of the question, we believe that Rgf1 is essential to mark the pole only in the absence of Tea1/Tea4, because these cells (*tea1Δ*) already have problems to find their ends (they are monopolar). In the presence of polarity markers, *rgf1Δ* cells are monopolar but they do not form Ts. It has to be both, the actin and the canonical polarity markers "under minimum" for the cells to make a high number of Ts.

The main message of the paper is that Rgf1 forms a second pathway to define sites of growth in parallel to the Tea1/4 pathway delivered by microtubules. The main argument for this model is that double mutants of tea1Δ rgf1Δ form T-shaped cells even when not stressed by overgrowth, osmotic or heat treatments, conditions known to transiently depolarize the actin cytoskeleton and lead to formation of T-shaped cells in tea1/4Δ cells upon growth re-initiation. The authors interpret this finding as Rgf1 (acting through activation of Rho1) forming a second pole-marking pathway dependent on the actin cytoskeleton. I think it is equally possible (in my view quite likely) that the reason for the phenotype of rgf1Δ tea1Δ is that cells lacking Rgf1 experience transient loss of polarization, which acts is much the same way as other stresses that cause transient actin depolarization. This is a question that needs to be addressed, for instance by examining when tea1Δ rgf1Δ double mutant cells form Ts: Is it at random times? Or does growth stop because of low Rho1 activity (such as upon a CW failure that is successfully repaired) in

rgf1Δ and therefore this acts as a stress? This will allow to establish whether rgf1Δ acts as a stressor, or whether Rgf1 functions in parallel to Tea1.

Answer:

To address the editor's concerns, we examined when *tea1Δ rgf1Δ* double mutant cells form Ts using time-lapse microscopy (Movie S3). We have not observed any growth stops or a visible despolymerization of actin at a concrete moment, suggesting that the loss of Rgf1 does not act as a stressor *per se*. In addition, the SAP and CIP pathways are not constitutively activated in the absence of Rgf1.

Interestingly, we observed that T-shaped cell formation in the double mutant does not occur randomly but is preferentially seen after cell division. Usually the cell that inherits the non-growing end from the mother cell is unable to recognize or activate this old end. Consequently, the growth machinery organizes at a new site on the lateral side (see below). In other words, in the cell that inherits the non-growing end when it has to decide where to grow, actin does not polarize to the tip (inactive old end) and remains randomly distributed until it becomes concentrated on the lateral, giving rise to a new pole. This result could also explain why only 50% of the dividing cells form T-shaped cells, as only one of the two daughter cells (50%) inherits the monopolar pattern.

Page 19 and line 486, we have added a new movie (Movie S3) to show the growth pattern in *tea1Δ rgf1Δ* cells and a brief explanation of the movie.

Page 19 and line 487. "These cells usually fail to choose the growth site after division. Usually the cell that inherits the non-growing end from the mother cell is unable to recognize or activate this old end and the growth machinery is organized at a new site on the lateral side (Movie S3)".

S3 Movie (page 44, line 1122). "LifeAct-GFP dynamics and morphology in rgf1Δ tea1Δ cells. LifeAct-GFP (green) and DIC (grey) were superposed to follow actin localization and morphology changes during 120 minutes (each picture every 2.5 minutes) in rgf1Δ tea1Δ cells growth in YES medium at 30°C."

LA-GFP *rgf1Δ tea1Δ*

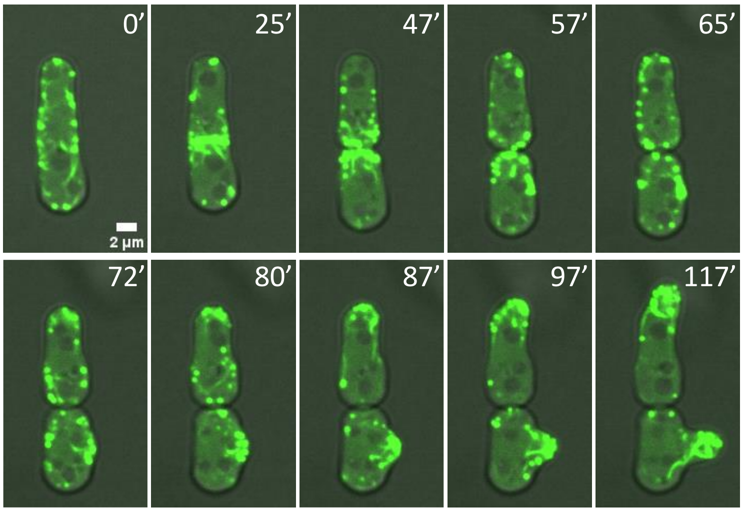

Beyond this issue on genetic interpretation of the double mutant phenotype, the Sawin group has recently shown that loss of F-actin does not lead per se to loss of polarity, nor polarized growth, but causes activation of the stress-activated MAPK pathway (SAPK; through the MAPK Sty1). This raises the question of whether all of the cases shown here to lead to T-shaped cells, and interpreted to act through the actin cytoskeleton, are not all due to activation of the SAPK (rather than F-actin depolymerization per se), including the rgf1Δ genotype. One simple experiment to start with is to probe whether branches form in tea1Δ rgf1Δ sty1Δ triple mutant. Further tools, for instance developed by the Sawin group, are available to test this hypothesis.

In summary, my view is that the paper has potential, but the main message is not (yet) well supported, and I would not be surprised if more detailed investigation reveals that rgf1Δ activates the SAPK, and that this causes the observed phenotypes.

We have addressed the editor's request regarding the role of the MAP kinase Sty1 pathway in the context of our study. Our findings indicate that the absence of Rgf1 does not act as a stressor that activates the Sty1 pathway. First, we observed no phosphorylation of Sty1 or the transcription factor Atf1 in the *rgf1Δ* mutant in basal conditions (Garcia et al 2009 and Fig S5H); second, there was no increase in the nuclear localization of Sty1 in *rgf1Δ* cells (Fig S5G). Moreover, the SAP pathway did not play a significant role in the polarity defects observed in the *rgf1Δ tea1Δ* double mutant. Specifically, the number of cells forming T shapes in the absence of both Rgf1 and Tea1 does not decrease upon the elimination of Sty1 (Fig S5J).

Here is the revised and expanded explanation for our findings:

In Page 21, line 521 it has been included the following paragraph

"Because stresses that induce actin disorganization and Rgf1 delocalization at the poles increase the percentage of T-shaped cells in the tea1Δ mutant (Fig 5D 5E, S5A and S5AS5B), we asked whether the absence of Rgf1 by itself acts as a stressor, thereby increasing the number of branched cells in the double mutant. In this regard, it has been demonstrated that treatment with LatA causes a delocalization of proteins necessary for polarized growth, such as the active form of Cdc42. The dispersal of Cdc42 from growth sites depends on the activation of MAP kinase Sty1 while it is independent of actin delocalization per se (52,54). Our group described years ago that while Rgf1 stimulates Pmk1 MAPK pathway activity in response to certain stresses, it was not involved in the activation of Sty1, neither under stress nor in basal conditions (38). Accordingly, we observed no increase in the nuclear localization of Sty1, nor in the phosphorylation of the transcription factor Atf1 in the rgf1Δ mutant (Fig S5G and H), as would be expected if Sty1 were active (55). Unlike delocalization of the active form of Cdc42 (marked by CRIB-GFP) (52,54), Rgf1 delocalization after stress does not depend on Sty1 (Fig S5I). Moreover, the number of cells forming T-shapes in the absence of both Rgf1 and Tea1 does not decrease upon the elimination of Sty1 (Fig S5J). Taken together, these results indicate that the absence of Rgf1 does not activate the Sty1 pathway (stress activated pathway, SAP), nor is the SAP important for the loss of polarity that occurs in the rgf1Δ tea1Δ double mutant.

An alternative explanation for the observed increase in branched cells when both Rgf1 and Tea1 were absent is that Tea1 and Rgf1 participate in parallel pathways to define the sites of cell growth, each one necessary when the other is not present. Thus, we wondered ….."

We have included Fig. S5G, H, I and J and the corresponding figure legends *(page 43, line 1095):*

"(G) GFP fluorescence in WT and *rgf1Δ* log-phase cells expressing *sty1-GFP.* Cells either were untreated (upper panels) or treated with 1 mM H_2O_2 for 15 min (lower panels). The maximum-intensity projection of six Z-slides (0.5 µm step-size) of Sty1-GFP fluorescence is shown. (H) Extracts from wild type (WT-unt) and *rgf1Δ* cells (*rgf1Δ*-unt) were compared with extracts from

WT cells treated with 1 mM H_2O_2 for 15 min. Proteins were visualized by western blot with antibodies against Atf1. (I) GFP fluorescence in WT and *sty1Δ* log-phase cells expressing *Rgf1-GFP*. Cells were either untreated (upper panels) or treated with 0.6 M KCl for 1 hour (lower panels). The maximum-intensity projection of six Z-slides (0.5 µm step-size) of Rgf1-GFP fluorescence is shown. (J) The percentage of T-shaped cells (n > 200) in the indicated strains grown to log phase in YES liquid medium at 28°C. Statistical significance was calculated using a two-tailed unpaired Student's t test. ns = non-significant. The experiment was repeated 4 times."

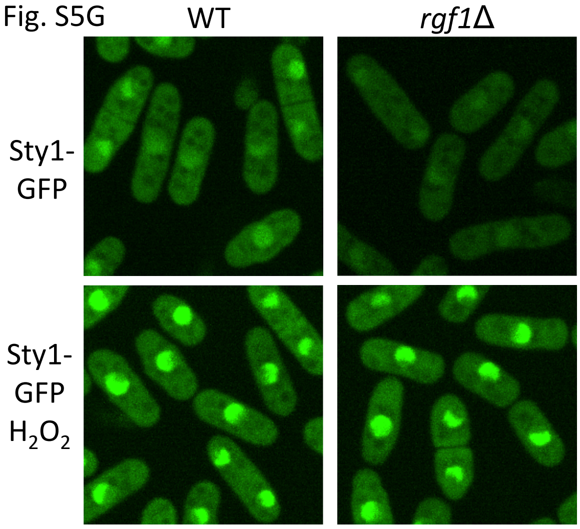

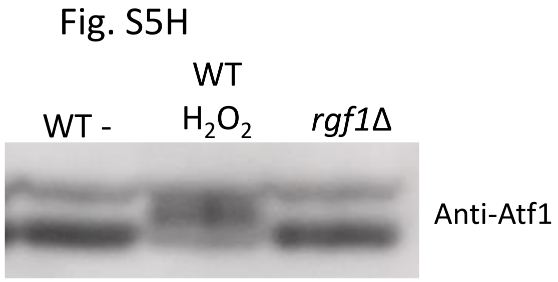

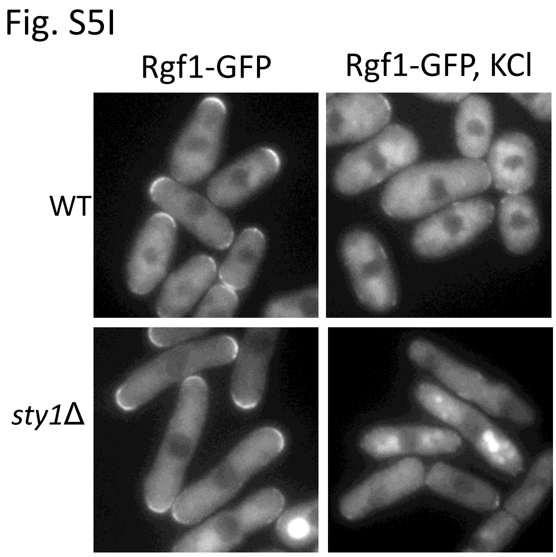

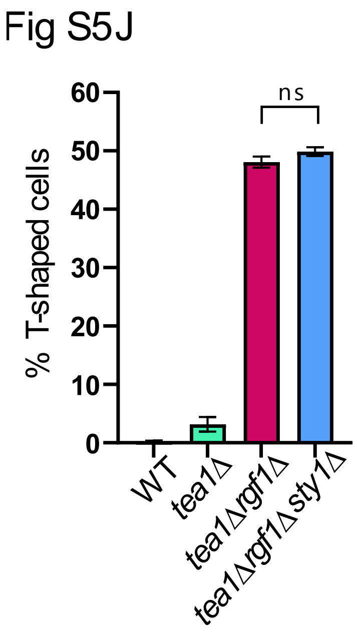

We hope that the new results added have resolved the editor's concerns regarding the role of Rgf1 in defining the sites of cell growth.
