# Supplementary material for: Rho1 and Rgf1 establish a new actin-dependent signal to determine growth poles in yeast independently of microtubules and the Tea1–Tea4 complex
Source: PLoS Biol. 2024 Nov 7;22(11):e3002491. doi: 10.1371/journal.pbio.3002491 (PMC11602027; doi:10.1371/journal.pbio.3002491)
Supplement: Attachment — Submitted filename: Response to reviewers, second revision.pdf [file pbio.3002491.s016.pdf]

PBIOLOGY-D-23-03415R1 (response to reviewers)

Rev. 1:

Thank you for the opportunity to assess the revised version of this manuscript. In my previous evaluation, I expressed concerns about the data presentation, experimental design, and quantification of several experiments, largely regarding the analysis of how Rgf1 contributes to the anchoring of Tea4 and Tea1 polarity markers at the pole. I am pleased to report that the authors have satisfactorily resolved most of these concerns in the revised manuscript. However, there are still some aspects that have been insufficiently developed in the current manuscript.

First, it is necessary to address the inconsistency in the findings regarding the effect of artificial targeting of Tea4 to the cell pole on the formation of T-shaped cells in the rgf1Δ mutant. The authors failed to provide an explanation for this discrepancy, and it is unclear why targeting Tea4 to the poles does not suppress the formation of T-shaped cells in this mutant. If the lack of anchoring of Tea4 at the poles results in a decrease in polarity markers and is the cause of the observed phenotype, it is expected that anchoring Tea4 to the poles would alleviate or prevent it.

ANSWER

We acknowledge that this experiment is not robust, and only a positive result will provide us with certainty. There are several ways to explain why the artificial targeting of Tea4 at the poles does not rescue the polarity defect observed in the *rgf1Δ* mutant in refeeding experiments. It is possible that Tea4 being permanently bound to CRIB at the poles is not fully functional; however, in that case, we would expect a higher percentage of T-shaped cells even without MBC, as occurs with the *tea1Δ rgf1Δ* double mutant (Fig. 5).

On the other hand, the fact that the defect is preferentially observed in cells treated with MBC leads us to think that the anchoring of Tea4 to the poles is likely insufficient to maintain Tea1 localized there as well. Therefore, in the absence of a continuous supply of Tea1 from the MTs (consequence of the MBC treatment), a situation similar to the *tea1Δ rgf1Δ* double mutant arises, where both pathways essential for the proper definition of the poles are not fully functional. Accordingly, we have included a paragraph in the discussion section to address this.

Page 23, line 597

However, while Tea4 correct localization is essential, simply targeting Tea4 to the cell poles is not sufficient to rescue growth site selection defects in the absence of Rgf1, especially when microtubules are disrupted by MBC treatment (Fig 4F). In this situation, Tea4-GBP-CRIB-GFP may be insufficient to maintain Tea1 localized to the pole as well. In cells treated with MBC, Tea1's absence at the poles could mimic the effects of a double mutant, *rgf1Δ tea1Δ*, because microtubules are necessary for the continuous supply of Tea1 to the poles. Without this fresh supply, even if Tea4 is localized correctly, its functional output might be compromised.

Second, I am not convinced of the representation of actin distribution data in rgf1Δ cells. Maybe the authors could use a more quantitative approach. In addition, the actin probe LifeAct-GFP,

driven by a strong actin promoter, has been shown to affect growth and division. I advise the authors to repeat experiments using high-resolution confocal microscopy and alternative and less detrimental means such as phalloidin staining, as in their original work, to validate these data.

ANSWER

Like any probe in a biological system, LifeAct-GFP is likely to influence actin assembly within cells. In *S. pombe*, the adverse effects from inappropriately stabilizing cellular F-actin structures were observed when LifeAct-GFP was expressed from the strong repressible P3Xnmt1 promoter {Courtemanche, 2016 #6445}. In contrast, the Life-Act-GFP construct we utilized came from M. Balasubramanian lab and was controlled by the actin promoter. In their study, it was demonstrated that cells expressing Life-Act-GFP displayed morphology, growth rate, and cell division similar to wild-type cells. Moreover, these cells exhibited no genetic interactions with temperature-sensitive mutants deficient in *cdc3*-profilin, *cdc8*-tropomyosin, or *cdc12*-formin {Huang, 2012 #5811}. The reviewer´s recommendation to use phalloidin staining also carries risks. Phalloidin binding to actin can disrupt the natural turnover of F-actin, potentially altering its interactions with actin-binding proteins (ABPs), particularly those involved in sensing the nucleotide state of actin for directed transport or cytoskeletal remodeling {Pospich, 2020 #6446}. Thank you for your suggestion, but according to the editor, the results presented are sufficient.

Nevertheless, my primary reservation about this work remains the insufficient demonstration of the mechanism by which Rgf1 acts redundantly in establishing polarized growth in fission yeast. My opinion on this aspect has remained unchanged. I convey that the main message of this study is that Rgf1 forms a second pathway dependent on the actin cytoskeleton to define sites of growth in parallel to the Tea1/4 pathway delivered by microtubules. However, I have concerns regarding the novelty of this observation and the extent to which this conclusion was developed in the current manuscript. That F-actin cooperate with Tea1-Tea4 complex in the establishment of polarity was already published in Cell by Damian Brunner lab in 2016 (10.1016/j.cell.2016.04.037). This study extends this observation by investigating Rgf1 as an activator of Rho1.

ANSWER

Respectfully, we disagree, in the study published by the Brunner lab in 2006, they focus on the role of sterol-rich membrane (SRM) domains in polarity during starvation exit. Regarding actin, they show that actin is important for removing unwanted SRMs from the central areas of the cell, which could serve to prevent growth at inappropriate locations, but they did not go further to study the mechanism of SRMs removal. In this study, they neither demonstrate nor suggest that actin is an alternative pathway to Tea1 for positioning the poles, nor do they link Rho1 and Rgf1 to this process at any point. Therefore, we believe our work is sufficiently novel.

More recently, a related study by Ribas et al. at the Instituto de Biología Funcional y Genómica (IBFG, Salamanca) was published in iScience (Ramos et al., iScience, 2024, Doi: 10.1016/j.isci.2024.110477). In this study, the authors propose a similar redundant pathway to Tea1-Tea4 in growth polarity establishment involving Bgs1 and actin patches. Both the absence of Bgs1 function and defective actin patch localization caused a similar total loss of growth polarity in the absence of the Tea1-Tea4 complex. This work suggests that Bgs1 is necessary for

the correct localization of actin patches and sterol-rich domain domains, which, in turn, are ultimately responsible for the control of growth polarity along with the Tea1-Tea4 complex. This raises the question whether Bgs1 is one of the targets of Rho1.

ANSWER

We became aware of this study after submitting the revised version of the manuscript. In this study, a redundant role between the glucan synthase Bgs1 and Tea1/Tea4 during polarized growth is indeed proposed. However, the double deletion of Bgs1 and Tea1 results in round cells, which is very different from the T-shaped cell phenotype observed in the *rgf1Δ tea1Δ* mutant. This indicates that the processes controlled by Bgs1 and Rgf1/Rho1 are likely different. Accordingly, we have included a paragraph (and the reference) in the discussion section to address this.

Page 25, line 633.

Recent findings also highlight the redundant role of the glucan synthase Bgs1 and polarity markers in polarized growth (57). The localization of actin patches and sterol-rich domains at the plasma membrane depends on Bgs1 function. However, when both the Tea1-Tea4 complex and Bgs1 were absent simultaneously, there was a complete loss of growth polarity. This resulted in cells taking on a spherical shape rather than the characteristic T-shaped morphology (consequence of loss polarity position) observed when Rgf1 and Tea1-Tea4 were lacking. This suggests that Bgs1 and Rgf1/Rho1 play distinct roles in regulating the polarity process.

Moreover, the authors did not provide sufficient evidence to explain the involvement of both actin patches and cables in the formation of T-shaped cells. This aspect has not been adequately addressed in this study. Furthermore, in the Discussion section, the authors posit that the activation of Rho1, rather than Cdc42, is responsible for defining the growth sites. However, this conclusion is based solely on the findings obtained using the gef1 mutant. The authors did not explore whether other GEFs, which are more relevant to Cdc42 activation during polarized growth, participate in this process. As a result, I believe that this assertion should be removed from the manuscript.

ANSWER

Ok, as the referee suggested we have removed the sentence. The paragraph has been modified as follow. Page 25, line 632.

Interestingly, in the absence of Tea1 and Gef1 (Cdc42 GEF), which is also involved in polarized growth and relies on actin for proper localization (52,53,56), cells do not form branches, suggesting that ~~is~~ the activation of Rho1 ~~and not that of Cdc42 which~~ specifically defines the growth sites in the absence of Tea1–Tea4.

Rev. 2:

I am satisfied with the authors' revisions. They performed a number of new experiments to address reviewer comments, I was particularly impressed with increased expression of the PH

mutant using the Pact1 promoter. This paper represents a nice contribution to the field and raises some interesting questions for the field to address in the future.

Thank you very much for your kind words.

Sincerely

Yolanda Sánchez
